# Single cell analysis of *M. tuberculosis* phenotype and macrophage lineages in the infected lung

**DOI:** 10.1084/jem.20210615

**Published:** 2021-07-22

**Authors:** Davide Pisu, Lu Huang, Vipin Narang, Monique Theriault, Gabrielle Lê-Bury, Bernett Lee, Agnes E. Lakudzala, David T. Mzinza, David V. Mhango, Steven C. Mitini-Nkhoma, Kondwani C. Jambo, Amit Singhal, Henry C. Mwandumba, David G. Russell

**Affiliations:** 1 Microbiology and Immunology, College of Veterinary Medicine, Cornell University, Ithaca, NY; 2 Microbiology and Immunology, University of Arkansas for Medical Sciences, Little Rock, AR; 3 Singapore Immunology Network, Agency for Science, Technology and Research, Singapore; 4 Malawi Liverpool Wellcome Trust Clinical Research Program, University of Malawi College of Medicine, Blantyre, Malawi; 5 Department of Clinical Sciences, Liverpool School of Tropical Medicine, Liverpool, UK; 6 A*STAR Infectious Diseases Laboratories, Agency for Science, Technology and Research, Singapore

## Abstract

In this study, we detail a novel approach that combines bacterial fitness fluorescent reporter strains with scRNA-seq to simultaneously acquire the host transcriptome, surface marker expression, and bacterial phenotype for each infected cell. This approach facilitates the dissection of the functional heterogeneity of *M. tuberculosis*–infected alveolar (AMs) and interstitial macrophages (IMs) in vivo. We identify clusters of pro-inflammatory AMs associated with stressed bacteria, in addition to three different populations of IMs with heterogeneous bacterial phenotypes. Finally, we show that the main macrophage populations in the lung are epigenetically constrained in their response to infection, while inter-species comparison reveals that most AMs subsets are conserved between mice and humans. This conceptual approach is readily transferable to other infectious disease agents with the potential for an increased understanding of the roles that different host cell populations play during the course of an infection.

## Teaser

Through scRNA-seq and ATAC-seq, Pisu et al. characterize specific lung macrophage subsets that either restrict or promote *M. tuberculosis* growth. Comparable macrophage populations are present in the human airways, and cells show evidence of epigenetic imprinting.

## Graphical Abstract

**Figure:**
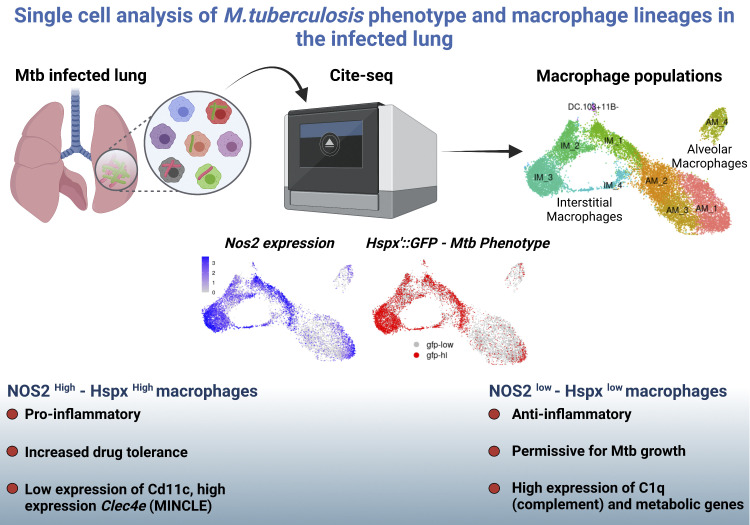

## Introduction

Fluorescent bacterial strains have been used in single-cell RNA-seq (scRNA-seq) studies probing host cell heterogeneity in vitro (Avital et al., 2017; Avraham et al., 2015; Saliba et al., 2016). However, in in vivo infections, where scRNA-seq as a discovery tool has considerably greater potential, the diversity of host cell types, the scarcity of infected cells, and the absence of information regarding the fitness of the infecting agent all pose challenges to interpretation of scRNA-seq datasets (Bost et al., 2020). These hurdles restrict the capacity of scRNA-seq studies to elucidate how different immune cells contribute to control or progression of infection. We sought to resolve these challenges in a murine model of tuberculosis through the use of a stress-inducible fluorescent bacterial fitness reporter in combination with flow cytometry and scRNA-seq from in vivo infection, to inform and empower our analytical pipeline.

*Mycobacterium tuberculosis* (Mtb) remains the greatest cause of death by a single infectious agent and is calculated to have a penetrance extending to 23% of the human population (Houben and Dodd, 2016). In immune-competent individuals, the parameters that determine control or progression of disease remain extremely poorly defined. Macrophages represent the most significant infected host cell and were regarded as a homogenous, blood monocyte–derived cell lineage that was programmable by cytokines to adopt differing activation states (van Furth and Cohn, 1968). However, fate-mapping and cell-profiling studies have shown that macrophages resident in tissues, such as the lung and skin, arise from various stem cell lineages during embryonic development (Gibbings et al., 2017; Ginhoux and Guilliams, 2016; Ginhoux and Jung, 2014), in addition to those cells recruited from the blood. To date, two main macrophage populations have been identified in the lung: tissue-resident alveolar macrophages (AMs) and monocyte-derived interstitial macrophages (IMs). Recent work, including our previous studies, has started to shed light on the role of these different macrophages lineages in Mtb infection in vivo (Huang et al., 2018), revealing that AMs constitute an anti-inflammatory M2-type population whose environment is favorable for Mtb replication and dissemination (Cohen et al., 2018; Huang et al., 2018; Pisu et al., 2020a), while IMs are associated with an immune milieu more stressful for the bacteria (Huang et al., 2018; Pisu et al., 2020a). However, these studies lack the ability to resolve the functional heterogeneity known to exist within these two main macrophage lineages (Chakarov et al., 2019; Evren et al., 2021; Liu et al., 2019).

Building on the studies of Stoeckius et al. (2017), we developed a multimodal approach to associate bacterial and host cell phenotypes at the single cell level. Using a bacterial reporter strain whose fluorescent expression correlates with the amount of environmental stress sensed by Mtb in each individual host cell (Abramovitch et al., 2011; Sukumar et al., 2014; Tan et al., 2013; Tan et al., 2017), we were able to simultaneously acquire the host transcriptome, surface markers expression, and the bacterial fitness phenotype. Through data integration (Korsunsky et al., 2019), we identified macrophage populations with common cell identities across different infection states. This enabled us to characterize those AM subsets that either restricted or promoted bacterial growth, in addition to defining a population of replicating tissue-resident AMs. We also identified three distinct populations of IMs: a population of monocyte-derived erythrophagocytic macrophages with high levels of *Nos2* expression that induced drug tolerance in Mtb; a population of anti-inflammatory Nrf2-expressing IMs associated with bacteria sensing a low amount of environmental stress; and finally a population of *Zeb2*-expressing IMs involved in resolution of inflammation. Moreover, comparison between the mouse macrophage subsets and the scRNA-seq profiles from bronchoalveolar lavage (BAL)–derived human lung macrophages indicated that the major subsets were present in both species. Finally, to assess the extent to which the responses of lung macrophages to mycobacterial infection are epigenetically determined, we performed transposase-accessible chromatin sequencing (ATAC-seq) analysis of both IM and AM populations from mice following i.v. inoculation with live *Mycobacterium bovis* bacillus Calmette–Guérin (BCG). These data suggest that much of the divergence of response between AMs and IMs is epigenetically controlled and actually precedes mycobacterial insult. The multimodal scRNA-seq approach detailed here is readily transferable to other infectious disease models with the potential to improve our understanding of the impact that the innate immune environment exerts over the outcome of infection. An appreciation of how host cell populations are regulated before and following infection is critical to the challenges facing vaccine development, as well as minimizing both drug tolerance and acquisition of drug resistance.

## Results

### Analysis of the scRNA-seq dataset

**Figure 1.**
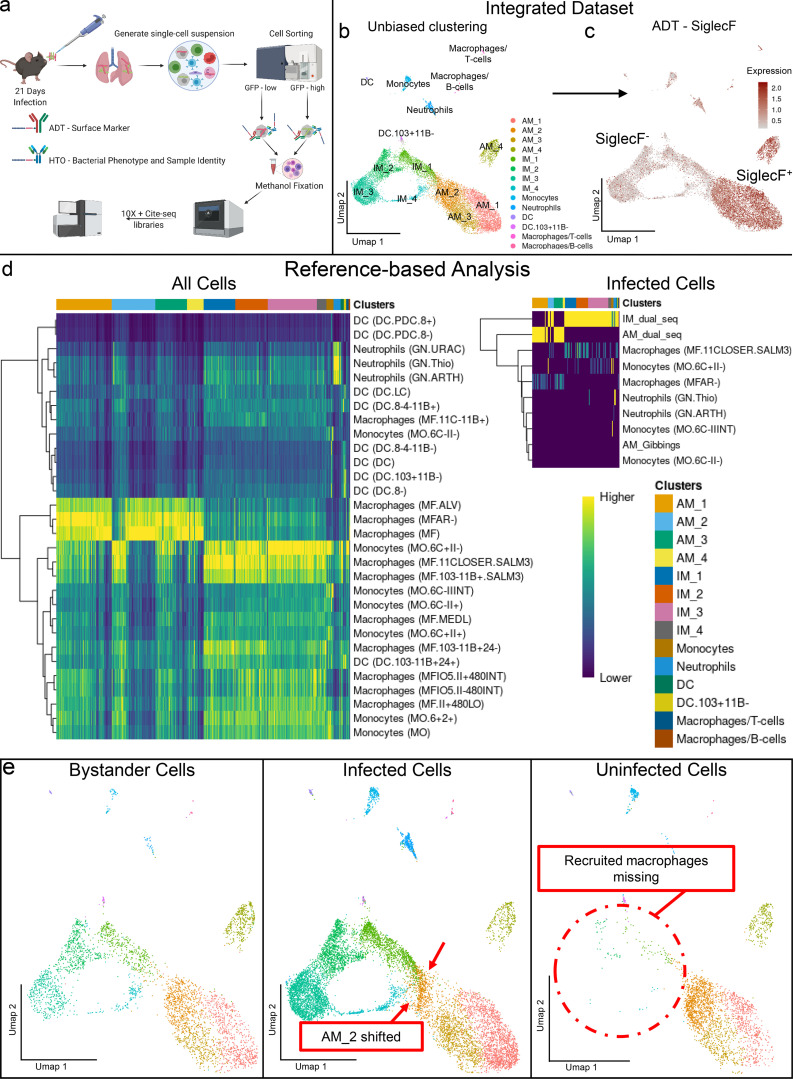
**Identification of heterogenous macrophage populations by scRNA-seq in Mtb-infected mouse lungs.** Here we show the results from unbiased analysis of the integrated myeloid datasets, leading to the identification of different subsets of infected AMs and IMs. **(a)** Schematic representation of the experimental protocols. Bl/6 mice were infected with either *hspx′*::GFP/*smyc′*::mCherry or *smyc′::mCherry* Erdman for 3 wk. Single-cell suspensions were generated and infected (mCherry^+^, *hspx′*::GFP^high/low^), bystander (mCherry^−^ CD45^+^), and uninfected (CD45^+^) cells were flow-sorted. Samples have then been stained with HTO (sample identity) or ADT (surface markers expression) antibodies and methanol-fixed, and 10X Genomics/Cite-Seq libraries were generated. **(b)** Umap plot showing unbiased clustering of the myeloid cell populations in the integrated dataset. All statistically significant (as defined by the jackstraw method; Chung and Storey, 2015) PC components have been used for clustering and downstream analysis. **(c)** Umap plot showing ADT staining levels (in log-normalized values) for the SiglecF protein. Two main macrophage populations, SiglecF^+^ and SiglecF^−^, are identifiable. Higher staining values are displayed in dark red, while lower staining values are shown in gray. **(d)** Heatmaps showing the results of reference-based analysis for either all cells in the integrated dataset against the Immgen database alone, or infected cells against a custom joined reference dataset that includes both general populations (Immgen) and Mtb-infected and uninfected lung AMs and IMs (Gibbings et al., 2017; Pisu et al., 2020a). Higher similarity scores among the transcriptional profiles of query and reference datasets are showed in yellow. **(e)** Umap plot showing a split view (based on infection status) of the unbiased clustering of the myeloid cell subsets. As described in the main text, recruited myeloid cells are absent from uninfected mice, while AM_2 cells are shifted and close to the IM_1 subset in the infected population. *n* = 5 for the infected population, *n* = 2 for the uninfected population.

**Figure S1.**
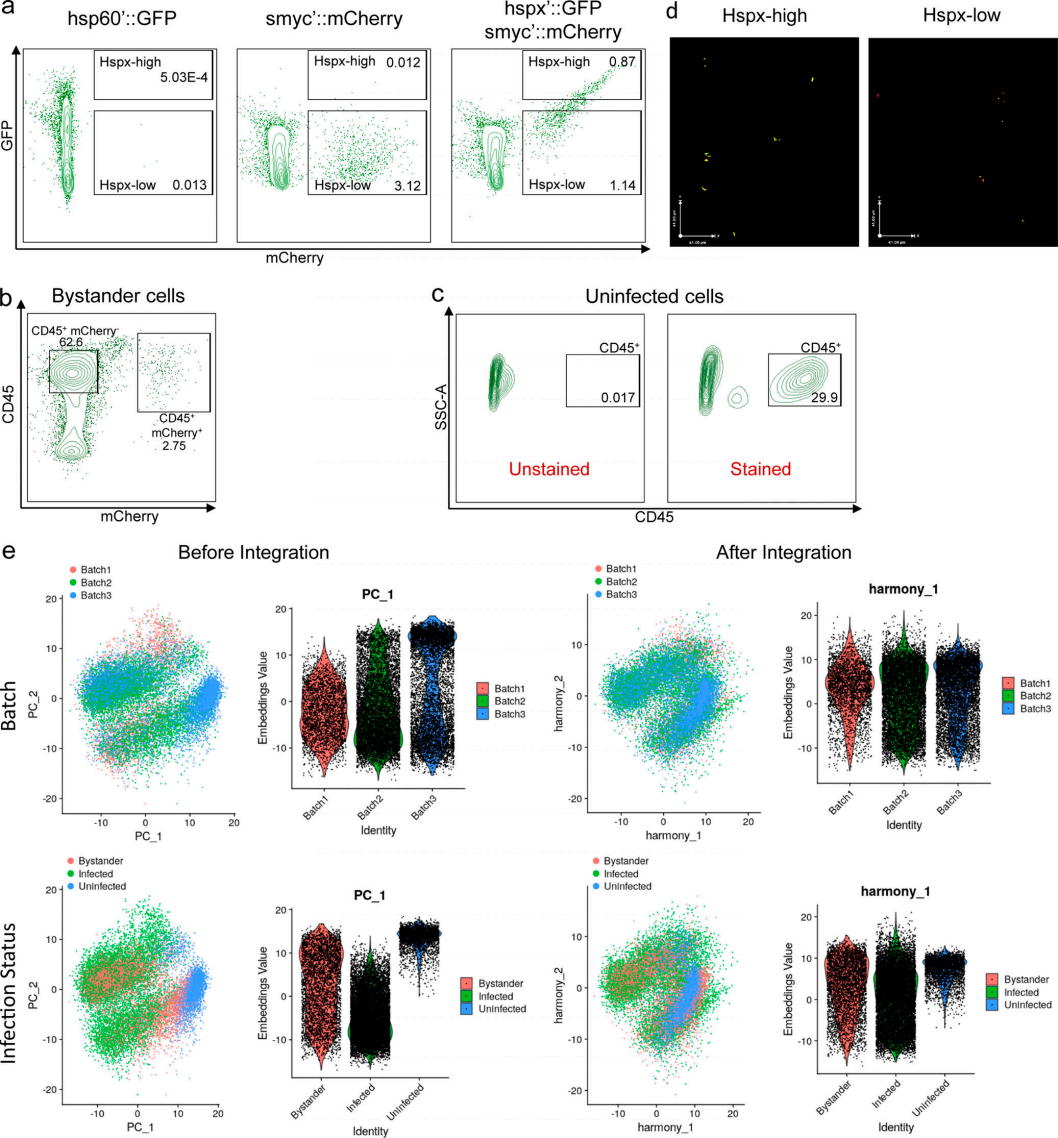
**Flow cytometry gating and scRNA normalization strategies used in the generation of the integrated dataset. (a)** Flow cytometry gating strategy for the sorting of the *hspx′*::GFP/*smyc′*::mCherry infected mice lungs. *hspx′*::GFP^high^ and *hspx′*::GFP^low^ infected host populations have been sorted based on the level of expression of a bacterial protein (Hspx), which is a well-known indicator of bacterial stress responses in Mtb. Mice infected with *′*::GFP Erdman (constitutive expression of GFP driven by *hsp60* promoter) and *smyc′*::mCherry (constitutive expression of mCherry driven by *smyc′* promoter) have been used as gating controls. **(b)** Flow cytometry gating strategy for the sorting of the bystander cells. mCherry^−^/CD45^+^ cells from mice infected with *smyc′*::mCherry Erdman have been sorted and processed for scRNA-seq. A mouse infected with WT Erdman (not fluorescent) has been used as a gating control for the mCherry signal (data not shown). **(c)** Flow cytometry gating strategy for the sorting of the uninfected cells. CD45^+^ cells from uninfected mice have been sorted and processed for scRNA-seq. An unstained sample has been used as a gating control. SSC-A, side scatter area. **(d)** Confocal microscopy images of the sorted populations: *hspx′*::GFP^high^ and *hspx′*::GFP^low^. **(e)** PCA and violin plots visualizing the merged myeloid datasets before and after integration with Harmony for the two covariates: infection status and batch. As visualized in the figure, data integration with Harmony allowed us to correctly identify shared cell identities from cells of different batches and infection statuses. *n* = 5 for the infected population, *n* = 2 for the uninfected population.

To acquire a functional appreciation of the macrophage subsets present in the Mtb-infected mouse lung, we developed a multimodal scRNA-seq protocol that allowed us to integrate bacterial phenotype with scRNA-seq data (Fig. 1 a). We infected mice for 21 d with an Mtb reporter strain (*hspx′*::*gfp*/*smyc′::mCherry*) that enabled us to sort infected host cells based on the level of GFP fluorescence expressed by intracellular Mtb. The Mtb GFP expression is driven by the *hspx* promoter, which is responsive to DosR, the major stress regulon for Mtb (Sukumar et al., 2014; Fig. S1, a and d). We tagged both populations of Mtb-infected cells (*hspx′*::GFP^high^ and *hspx′*::GFP^low^) with different hashtag oligos (HTOs) for sample identity but with a common set of antibody-derived tags (ADTs) for surface marker expression (Fig. 1 a). Bystander (CD45^+^mCherry^−^ from infected mice) and uninfected cells (CD45^+^ from uninfected mice) were also sorted and processed for scRNA-seq (Fig. S1, b and c). This allowed us to integrate transcriptomic information with surface marker expression and the associated bacterial phenotype of the immune cell populations. Analysis of the scRNA-seq datasets from infected cells revealed that ∼80% were of myeloid origin (Table S1), of which >90% were macrophages, which constitute the major focus of our analysis.

Using Harmony (Korsunsky et al., 2019), we integrated the myeloid populations across all datasets, to identify common cell identities and to normalize for batch effects and infection status (Fig. S1 e). A total of 17,101 myeloid cells were recovered across all datasets, and unbiased clustering was performed using Seurat in R (Fig. 1 b). We previously defined the two ontologically distinct populations of Mtb-infected lung macrophages, AM and IM, based on the expression levels of SiglecF, with AM being CD64^+^MerTK^+^SiglecF^+^ and IM CD64^+^MerTK^+^SiglecF^−^ (Huang et al., 2018; Pisu et al., 2020a). ADT against SiglecF confirmed the identities of AM and IM (Fig. 1 c). Using the Immunological Genome Project (Immgen) database (Heng et al., 2008), we performed reference-based analysis (Aran et al., 2019) of our integrated dataset to assign cellular identities. The results clearly indicate the presence of a group of cells defined as AMs (MF.ALV and MFAR^−^) and a more heterogenous population of recruited macrophages (Fig. 1 d). To further validate the infected macrophage populations, we used transcriptional data from previous studies (Gibbings et al., 2017; Pisu et al., 2020a) to generate a custom joined reference, as previously described (Aran et al., 2019). We show that both AMs and IMs in our scRNA-seq dataset correlate with the infected cell populations detailed previously (Fig. 1 d).

**Figure S2.**
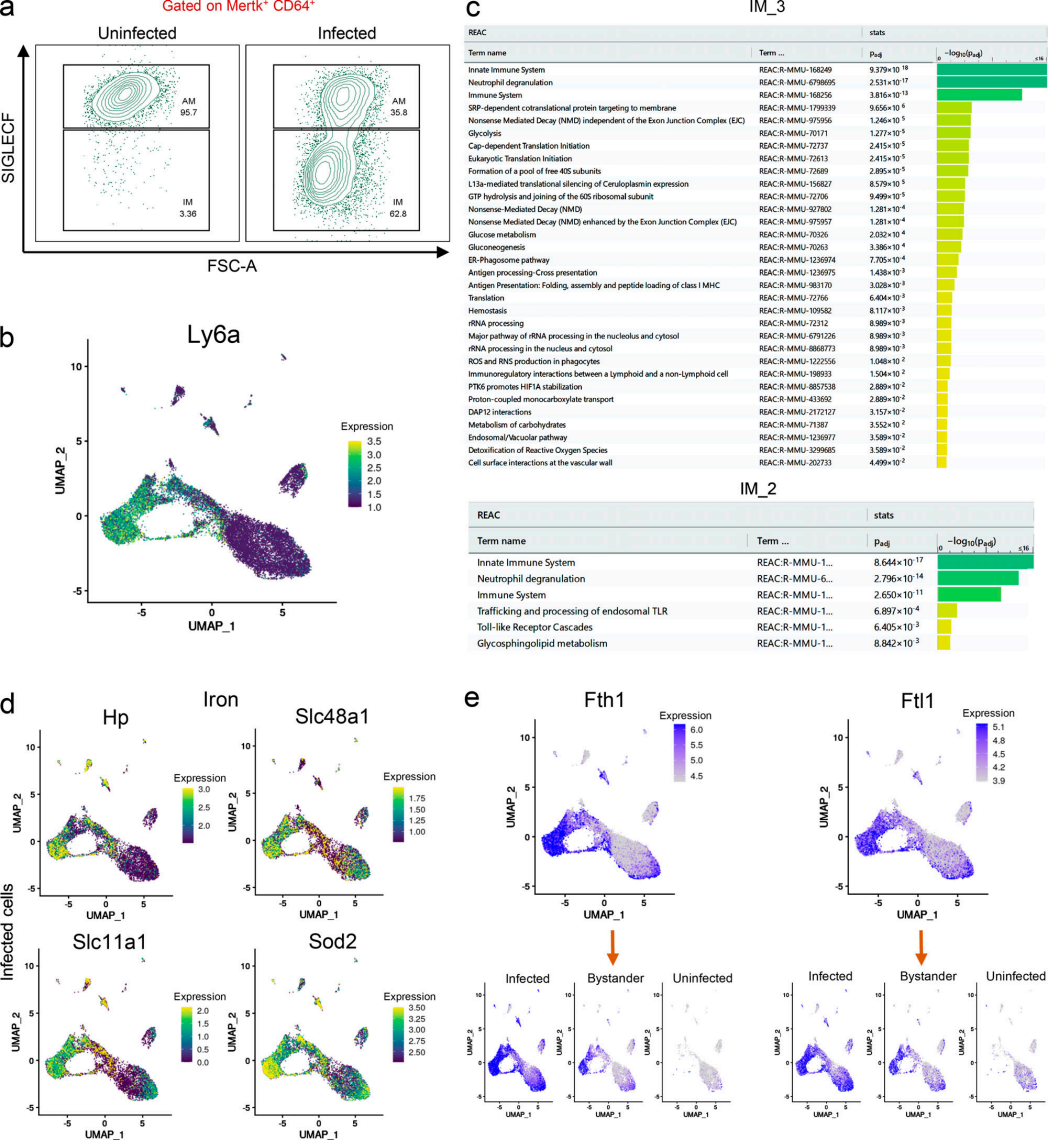
**Pathway enrichment analysis and assessment of genes associated with an iron signature for the IM_2 and IM_3 subsets. (a)** Flow cytometry analysis of the two ontologically distinct macrophage populations from infected (at 3 w.p.i.) and uninfected mice lungs. Macrophages (MerTK^+^ CD64^+^) have been gated from other immune cells, and expression of the surface marker SiglecF has been used to separate the two main populations (AMs and IMs). As evidenced in the text, recruited populations of macrophages are present in lower numbers in uninfected mice compared with infected tissues. FSC-A, forward scatter area. **(b)** Umap plot showing expression levels (in log-normalized counts) for the *Ly6a*/Sca-1 marker gene. IM_2 and IM_3 subpopulations both express this marker, although only IM_3 shows a pro-inflammatory phenotype. Higher expression values are displayed in yellow/green, while low expression values are shown in blue. **(c)** Pathway enrichment analysis results for the transcriptional profile of IM_3 and IM_2 infected macrophages. The reactome database has been used as the main data source. **(d)** Umap plots showing gene expression levels (in log-normalized counts) for some of the genes (*Hp*, *Slc48a1*, *Sod2*, and *Slc11a1*) involved in the heme-iron response in IMs. Higher expression values are displayed in yellow/green, while low expression values are shown in blue. **(e)** Umap plots (split-view) showing gene expression levels (in log-normalized counts) for the ferritin genes (*Ftl1* and *Fth1*) across different infection conditions. Higher expression values are shown in blue, while low expression values are displayed in gray. *n* = 5 for the infected population, *n* = 2 for the uninfected population. All the genes highlighted in the different figures show statistically significant greater expression in their clusters compared with cells in different clusters (FDR < 0.05; Wilcoxon rank-sum test; see Materials and methods). For pathway enrichment analysis, only pathways with FDR < 0.05 are shown (g:SCS method for multiple testing correction; See Materials and methods).

The majority of IMs are recruited from the peripheral blood to the infection site in the lung (Huang et al., 2018). Analysis of the shared cell states from the integrated dataset confirms previous observations that the number of IMs present in the uninfected mice lung is markedly less than in infected tissue (Fig. 1 e), which is in agreement with flow cytometry analysis (Fig. S2 a).

### Integrating bacterial and immune phenotypes in scRNA-seq defines macrophage subsets associated with *hspx′*::GFP^high^ Mtb

**Figure 2.**
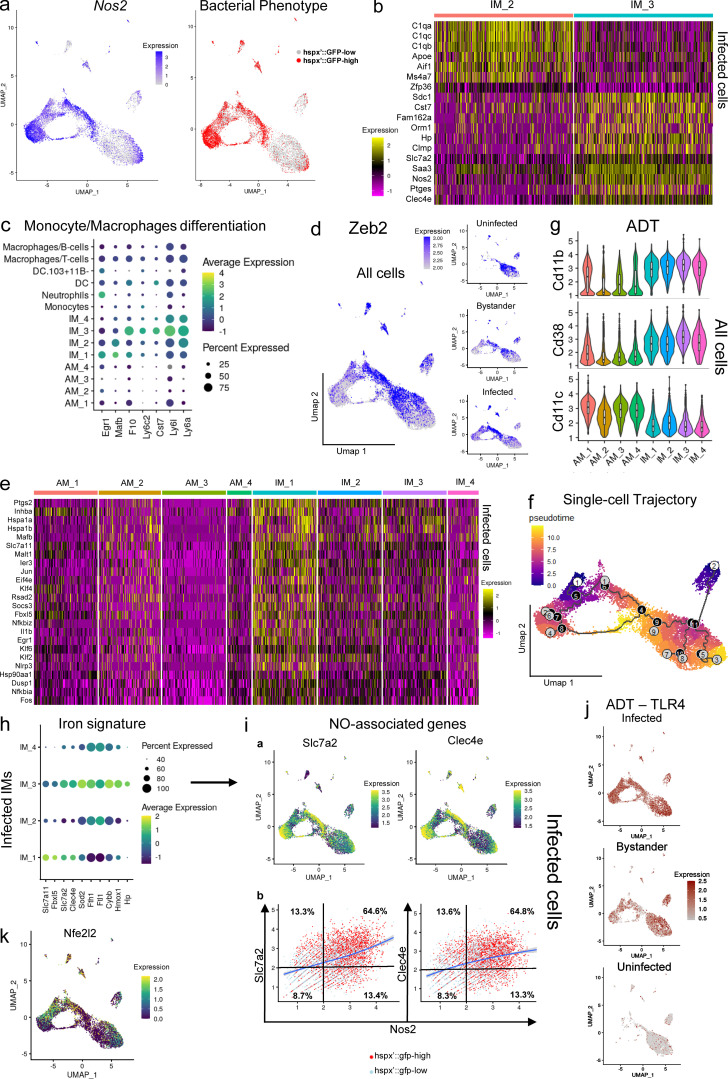
**Analysis of the IM subsets with respect of their impact on bacterial phenotype. **Here we probe deeper into the transcriptomes of those IM subsets that appear best adapted to control Mtb growth. **(a)** Umap plots showing a side-by-side comparison of the RNA expression levels for *Nos2* (in log-normalized counts) and associated bacterial phenotype across infected host cells. The data demonstrates that up-regulation of *Nos2* in infected cells (highlighted in blue) is associated with stressed bacteria (*hspx′*::GFP^high^, in red). **(b)** Heatmap showing the different gene signatures that characterize IM_2 and IM_3 subsets in infected cells. Higher expression levels are shown in yellow, lower expression levels in purple. Each row represents a gene, while each column represents a cell. **(c)** Dot plot showing scaled expression values for monocyte to macrophage differentiation marker genes among the different clusters of the integrated dataset. As evidenced in the text, the IM_3 subset shows the highest levels of expression of monocyte markers, suggesting that these cells are not yet fully differentiated into macrophages. Higher expression levels are represented in yellow/green, while lower expression levels are shown in blue. The size of the dot corresponds to the percentage of cells that express each gene in each cluster. **(d)** Umap plots (split-view) showing expression levels (in log-normalized counts) for *Zeb2* among the different infection conditions. IM_1 and AM_2 are the subsets characterized by the expression of this marker gene. Cells with high levels of expression are highlighted in blue. **(e)** Heatmap showing scaled gene expression levels in the AM and IM clusters for the transcriptional signature that characterizes the IM_1 subset. Higher expression levels are shown in yellow, lower expression levels in purple. Each row represents a gene, while each column represents a cell. **(f)** Umap plot showing single-cell trajectory and pseudotime analysis of the macrophage populations in the integrated dataset. Gray circles represent cell fates, while black circles are defined as branching points through which cells transition in the trajectory. For pseudotime distance, cells farthest away from the root are colored in yellow, while cells close to the root are colored in blue. **(g)** Stacked violin plots showing staining levels (in log-normalized values) for the ADT antibodies CD38, CD11b, and CD11c across IM and AM clusters of the integrated dataset. In general, IM subsets are characterized by higher staining levels for CD38 and CD11b antibodies, while expression of the surface marker CD11c is higher in AMs. **(h)** Dot plot showing scaled expression values for the iron gene signature among the infected IM subsets. Higher expression levels are represented in yellow/green, while lower expression levels are shown in blue. The size of the dot corresponds to the percentage of cells that express each gene in each cluster. **(i)** a: Umap plots showing expression levels (in log-normalized counts) for the *Slc7a2* and *Clec4e* genes, involved in NO-mediated responses to infection in macrophages. Cells with high levels of expression are displayed in yellow. b: Scatter plots showing coexpression levels of *Nos2* with either *Clec4e* and *Slc7a2* genes (in log-normalized counts) and associated bacterial phenotype for each cell (*hspx′*::GFP^high^ and *hspx′*::GFP^low^ in red and light blue, respectively). A subjective threshold of 2 log-normalized counts has been chosen to define high and low populations based on prior analysis of the distribution of expression for each gene among the different macrophage populations (a and i, top panel a). Percentage values indicate the amount of *hspx′*::GFP^high^ cells in each quadrant from the entire population of *hspx′*::GFP^high^ cells displayed in the plot. **(j)** Umap plots (split-view) showing ADT staining values for the TLR4 surface marker (in log-normalized counts) across the different infection conditions. Higher staining values are displayed in dark red, lower staining values in gray. **(k)** Umap plot showing expression values (in log-normalized counts) for the *Nfe2l2* (Nrf2) gene. The plot shows that this NO-associated gene is mainly expressed by IM_2 macrophages, as evidenced in the text, and some subpopulations of AMs, as previously described (Rothchild et al., 2019). Higher expression values are displayed in yellow, and lower expression values are shown in blue. *n* = 5 for the infected population, *n* = 2 for the uninfected population. All the genes highlighted in the different figures show statistically significant greater expression in their clusters compared with cells in different clusters (FDR < 0.05; Wilcoxon rank-sum test; see Materials and methods).

We superimposed our preliminary map of macrophage cell types with the expression of *hspx′*::GFP as an indicator of Mtb stress (Sukumar et al., 2014). Our analysis reveals that *hspx′*::GFP is expressed mainly by Mtb in *Nos2*^+^ macrophages (Fig. 2 a). Focusing on the IM clusters, we observe that the IM subpopulations IM_2 and IM_3 are associated with contrasting bacterial phenotypes. Only 39% of infected IM_2s contain *hspx′*::GFP^high^ bacteria, in comparison with 75% of the infected IM_3s (Table 1). Comparison of the transcriptional profiles of the two IM subsets reveals distinct gene signatures (Fig. 2 b). IM_2 macrophages express transcripts for complement proteins *C1q*, apolipoprotein *ApoE*, *Aif1*, *Ms4a7*,**and *Zfp36*, which were previously shown to be associated with M2 polarization and anti-inflammatory responses (Baitsch et al., 2011; Bohlson et al., 2014; Cai et al., 2017; Mattiola et al., 2019; O’Neil et al., 2017), while IM_3 macrophages exhibit a marked M1 inflammatory response including expression of transcripts for Mincle (macrophage-inducible C-type lectin, *Clec4e*) involved in direct recognition of mycobacterial cell wall lipids (Ishikawa et al., 2009), *Nos2*, *Saa3* (serum amyloid A), *Sdc1*, *Slc7a2*, and *Orm1* (Meek et al., 1992; Wells et al., 2008; Yeramian et al., 2006; Table S2, Table S3, and Table S4). A similar population of immature myeloid cells (*Ly6a*/Sca-1^+^), producing high levels of inflammatory molecules and with increased expression of monocyte-associated markers, was described in *Staphylococcus aureus* infection (Park et al., 2020). We found that both IM_2 and IM_3 populations are *Ly6a*/Sca-1^high^ (Fig. S2 b and Fig. 2 c); however, only IM_3 shows increased expression of pro-inflammatory genes (Fig. 2 b). Both reference-based (Fig. 1 d) and canonical marker analyses of our dataset (Fig. 2 c) suggest that this population of infiltrating myeloid cells (IM_3) exhibits a transcriptional profile that is intermediate between monocytes and macrophages. Intriguingly, pathway enrichment analysis confirms that up-regulation of glycolysis, nonsense-mediated mRNA decay, hemostasis, ROS, and reactive nitrogen species production are the dominant signatures of the IM_3 subset, while Toll-like receptor cascades and glycosphingolipid metabolism are up-regulated in IM_2 (Fig. S2 c). Our scRNA-seq data are consistent with our observations that a metabolic shift toward glycolysis is an hallmark of infected IMs (Pisu et al., 2020a).

**Table 1.** Absolute numbers and percentages of *hspx'*::GFP^high^ and *hspx'*::GFP^low^ Mtb present in each of the different macrophage subpopulations

Clusters	Total cells	Gfp-high	Gfp-low	% Gfp-High	% Gfp-Low
IM-1	1,396	668	728	48%	52%
IM-2	1,401	546	855	39%	61%
IM-3	2,436	1,837	599	75%	25%
IM-4	385	222	163	58%	42%
AM-1	1,868	253	1,615	14%	86%
AM-2	778	400	378	51%	49%
AM-3	1,005	85	920	8%	92%
AM-4	309	40	269	13%	87%

IM_1 macrophages are associated with a mixed bacterial phenotype (Table 1) and show lower expression of monocyte markers (Fig. 2 c). Intriguingly, these cells constitute a small population already present in the lungs of uninfected mice (Fig. 1 e). *Zeb2,* which is required to maintain tissue-specific macrophage identities (Scott et al., 2018), is overexpressed by this cluster of IMs (in both infected and bystander populations; Fig. 2 d). This subset is characterized by overexpression of cyclooxygenase2 (*Ptgs2*/Cox2) and *Il1β* (IL-1β), a pathway known to promote resistance in Mtb-infected mice (Mayer-Barber et al., 2014). The transcriptional profile of IM_1 macrophages is consistent with IL-17a–mediated inflammatory responses (Chen et al., 2013), with up-regulation of many genes that are involved in feedback inhibition and resolution of inflammation (*Nfkbia*, *Nfkbiz*, *Ier3*, *Hspa1a*, *Hspa1b*, *Hsp90aa*, *Vegfa*, *Ifrd1*, *Fos*, *Jun*, *Klf4*, *Klf6*, *Dusp1*, *Socs3*, and *Zfp36*; Schott et al., 2014; Fontana et al., 2015; Kapoor et al., 2015; Tummers et al., 2015; Kim et al., 2016; Wheeler et al., 2018; Fig. 2 e, Table S5, and Table S6). Consistent with this gene signature, the transcription factor *Atf3*, which has been shown to regulate ROS-dependent IL-1β activation and stimulate IL-17a responses in γδ T cells (Lee et al., 2018), and *Nlrp3* (Nalp3), whose expression leads to caspase 1 activation, pro–IL-1β, cleavage, and IL-1β secretion (Zhao et al., 2014), are also up-regulated in this sub-population of macrophages (Table S6).

Finally, IM_4s, which account for only 3.1% of the total cells in the dataset, are characterized by the expression of gene signatures in common with the IMs subsets described above (Table S7), suggesting that this cluster of cells may represent a transitional phenotype between the more defined populations. Trajectory analysis (Fig. 2 f) supports this hypothesis, as it shows that while IM_3s and a subset of IM_2s and IM_1s represent well-defined cell fates (gray circles), IM_4 is a cluster of cells transitioning from branching point 8 to 4 (black circles). Pseudotime analysis suggests that the direction of the movement is from IM_3 toward IM_1 (pro-inflammatory to resolution of inflammation).

**Figure S3.**
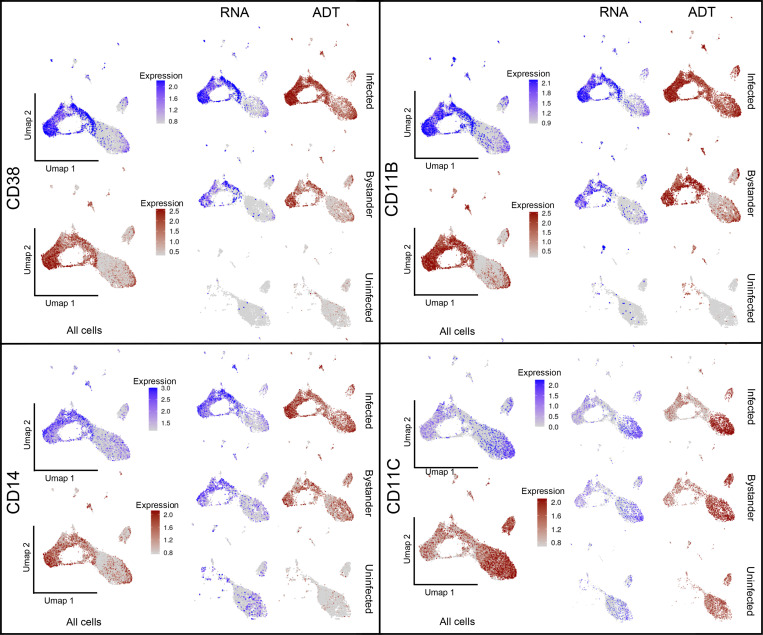
**Umap plots (split-view) showing both gene expression (RNA) and antibody staining levels (ADT) for the surface markers CD14, CD11b, CD11c, and CD38 across different infection conditions.***n* = 5 for the infected population, *n* = 2 for the uninfected population.

Consistent with previous work, ADT staining shows that IMs are characterized by increased expression of CD38, CD11b, and CD14 and decreased expression of CD11c (Fig. 2 g and Fig. S3). However, we note that the level of expression of these surface markers varies dramatically between the different infection conditions of the host cells, both at the protein and RNA levels (Fig. S3). Overall, our data indicate that the bulk IM population is comprised of several subsets that appear to transition between different states and assume different phenotypes with the potential to elicit divergent bacterial responses (see later sections).

### Diverging heme-iron responses are associated with different bacterial phenotypes in infected IM subsets

The host response to iron is well-documented as a factor of significance in Mtb infection (Amaral et al., 2019; Dixon et al., 2012; Haldar et al., 2014; Mitra et al., 2019; Phelan et al., 2018; Pisu et al., 2020a), and our scRNA-seq analysis reveals divergent and opposing signatures across the IM subsets.

IM_3 macrophages show increased expression of *Hp* (haptoglobin), known to control trafficking of hemoglobin to the lysosome (Asleh et al., 2014), the heme-responsive *Slc48a1* (Hrg1) involved in translocation of heme to the cytoplasm, the divalent metal transporter *Slc11a1* (Nramp1) involved in translocation of iron from phagolysosomes into the cytoplasm (Soe-Lin et al., 2009; White et al., 2013), and *Hmox1* (heme-oxygenase-1), which is induced upon accumulation of cytosolic heme (Gozzelino et al., 2010; Fig. 2 h and Fig. S2 d). ADT staining for TLR4 reveals that all infected IMs express TLR4 (Fig. 2 j). Signaling by pathogen recognition receptors (PRRs), such as TLR4, activates specific programs in macrophages associated with the production of ROS in a Nox2-dependent manner (Bedard and Krause, 2007; Yu et al., 1998). IM_3s overexpress both *Ftl1* and *Fth1* (the light and heavy chain of ferritin), indicating that iron storage is engaged to limit production of reactive radicals in this population (Fig. S2 e and Fig. 2 h). *Sod2* expression is also increased (Fig. S2 d). Overall, this transcriptional signature is usually associated with macrophages experiencing iron excess such as during erythrophagocytosis (Amaral et al., 2019; Soares and Hamza, 2016).

IM_2s share an iron-related transcriptional profile that is similar to IM_3, with overexpression of genes involved in iron removal from the phagolysosome (*Slc48a1* and *Slc11a1*) and iron storage (*Fth1* and *Ftl1*), suggesting that this is a common response at the site of Mtb infection (Fig. S2, d and e). However, they do not express *Hp* and show decreased expression of *Hmox1,* indicating that, compared with IM_3, heme iron accumulation is less prevalent in this population (Fig. 2 h and Fig. S2 d). IM_2s, but not IM_3s, express the transcription factor Nrf2 in infected cells (*Nfe2l2*), which is involved in regulation of heme-iron metabolism (Soares and Hamza, 2016; Fig. 2 k). Recently it has been reported that expression of a Nrf2-signature in Mtb-infected AMs leads to a decrease in pro-inflammatory pathways in early infection (Rothchild et al., 2019). Nrf2 activation in response to PRR signaling (TLR4) is known to be involved in a negative-feedback loop that results in inhibition of *Nos2* expression and nitric oxide (NO) production (Ashino et al., 2008; Gatbonton-Schwager et al., 2014). Here we show that while expression of Mincle (*Clec4e*) is absent from IM_2 macrophages (Fig. 2 i, top panel a), its expression correlates with *Nos2* and the *hspx′*::GFP^high^ bacterial phenotype in all other populations (Fig. 2, a and i, bottom panel b), implying that Mincle ligation in Mtb-infected macrophages may be necessary to maximize *Nos2* expression. Direct interaction of the hemeprotein NO with iron-containing components of the mitochondrial transport chain results in the metabolic switch to glycolysis (Kelly and O’Neill, 2015), a pathway up-regulated in IM_3, but not IM_2 (Fig. S2 c). A population of macrophages similar to the IM_2 cluster with high expression of C1q genes and genes involved in iron metabolism has also recently been reported in the lung macrophages of a humanized mouse model (Evren et al., 2021). Finally, our dataset reveals that expression of *Slc7a2*, a cationic amino acid transporter involved in arginine uptake, is up-regulated in macrophages infected with *hspx′*::GFP^high^ bacteria and overexpressing *Nos2* (Fig. 2 i), confirming previous observations (Yeramian et al., 2006) and suggesting that availability of arginine for NO production may also be a limiting factor in a sustained immune response against Mtb. This is consistent with the linear correlation in coexpression of both *Scl7a2* and *Nos2* (Fig. 2 i).

Among the genes most highly expressed by IM_1 macrophages are *Slc7a11* (xCT antiporter system), which has been shown to protect cells from iron-induced death (Dixon et al., 2012; Dixon et al., 2014) as well as *Fbxl5*, whose conditional deletion leads to iron overload and reduced cell numbers (Muto et al., 2019; Muto et al., 2017). In contrast, expression of *Fth1* and *Ftl1* is strongly down-regulated in this population, implying that, compared with IM_2 and IM_3, a different mechanism of action is involved in mitigation of ferroptosis and oxidative stress (Fig. 2 h and Fig. S2 e). Overall, our data provide further indications that host iron metabolism is interconnected to Mtb pathophysiology, and reinforce previous indications that immunometabolic reprogramming is integral to tuberculosis host defense and needs to be more effectively integrated with our therapeutic strategies (Palma et al., 2021; Phelan et al., 2018).

### AMs are characterized by subsets showing differing degrees of pro-inflammatory responses

**Figure S4.**
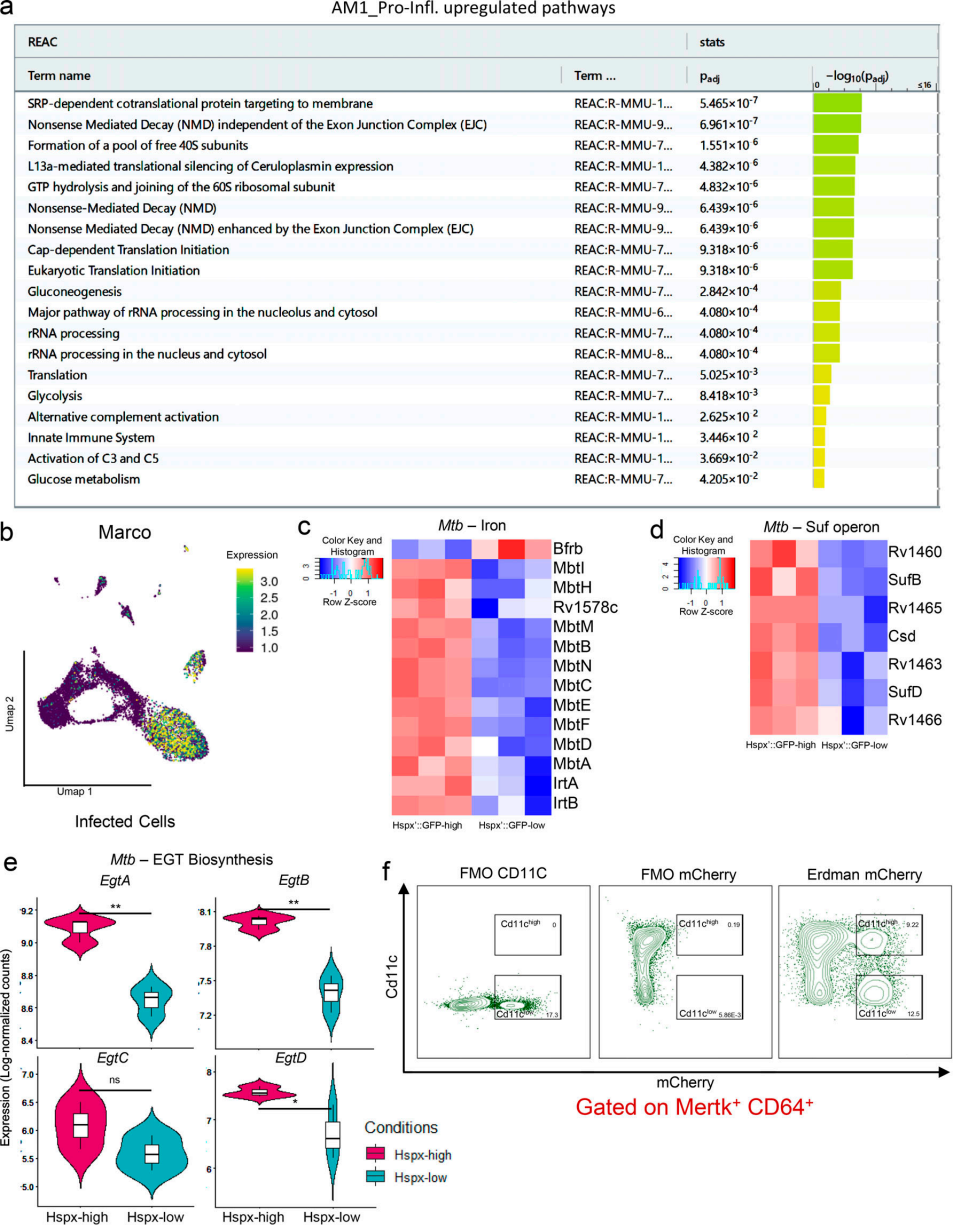
**AM1_Pro-infl subset gene signatures and Mtb transcriptional profile. (a)** Pathway enrichment analysis results for the transcriptional profile of the AM_Pro-Infl population. The reactome database has been used as the main data source. **(b)** Umap plot showing gene expression levels (in log-normalized counts) for the *Marco* PRR. Higher expression values are displayed in yellow/green, while low expression values are shown in blue. **(c)** Heatmap showing relative expression levels for the iron gene signature in Mtb. **(d)** Heatmap showing relative expression levels for the suf operon in Mtb. **(e)** Violin plots showing expression levels (in log-normalized counts) for the genes part of the ergothioneine biosynthesis pathway in Mtb. **(f)** Flow cytometry gating strategy for the sorting of CD11c^low^ and CD11c^high^ macrophage populations (MerTK^+^CD64^+^). Fluorescence Minus One (FMO) controls for both CD11c and mCherry signals have been used as gating controls. *n* = 5 for the infected population, *n* = 2 for the uninfected population, *n* = 3 for the bacterial transcriptome. The statistical significance for the genes part of the bacterial transcriptome has been calculated using the Wald test as implemented in the DESeq2 package (FDR < 0.05; see Materials and methods; Love et al., 2014). For pathway enrichment analysis, only pathways with FDR < 0.05 are shown (g:SCS method for multiple testing correction; see Materials and methods) Unless otherwise specified, the statistical significance is provided when appropriate for each plot (*, p-adj. < 0.05; **, p-adj. < 0.01).

**Figure 3.**
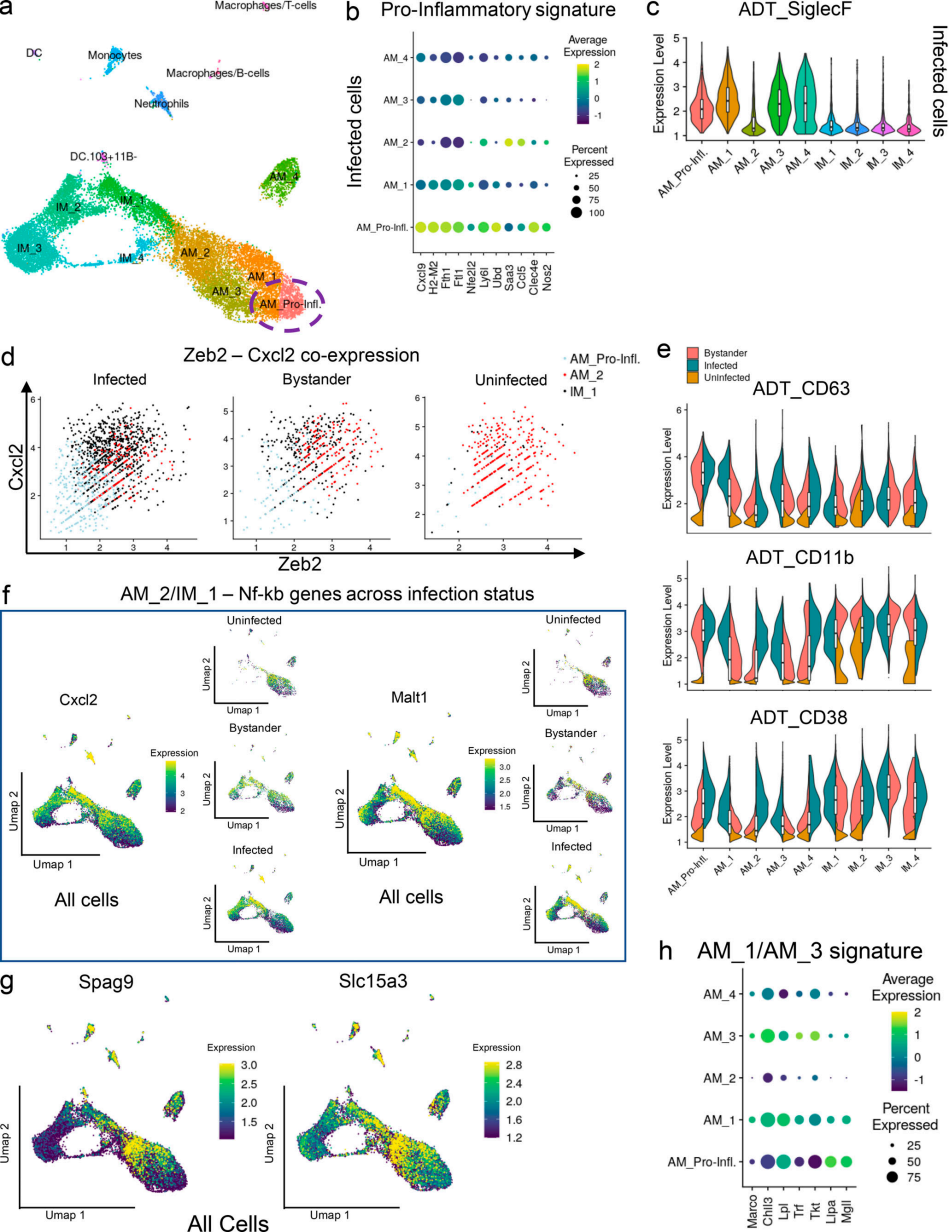
**Analysis of the pro-inflammatory AM subsets. **The AM subsets also include host cell populations that have an inflammatory phenotype and appear capable of inducing stress in Mtb.** (a)** Umap plot showing the clustering of the myeloid cell populations in the integrated dataset. The AM_Pro-Infl population, a subset of the AM_1 cluster, is highlighted. **(b)** Dot plot showing scaled expression values for the pro-inflammatory gene signature related to infected AMs. Higher expression levels are represented in yellow/green, while lower expression levels are shown in blue. The size of the dot corresponds to the percentage of cells that express each gene in each cluster. **(c)** Violin plot showing ADT staining levels (in log-normalized counts) for the SiglecF surface marker among the different subpopulations of infected macrophages. **(d)** Scatter plots (split-view) showing coexpression levels (in log-normalized counts) for *Zeb2* and *Cxcl2* marker genes in AM_2, IM_1, and AM_Pro-Infl subpopulations across different infection states. **(e)** Stacked violin plots showing staining levels (in log-normalized values) for the ADT antibodies CD63, CD11b, and CD38 in different clusters of macrophages and across the different infection conditions. As evidenced in the text, the infected and bystander AM_Pro-Infl populations show higher expression levels for these surface markers. **(f)** Umap plots (split view) showing expression values (in log-normalized counts) for the *Cxcl2* and *Malt1* genes (part of the AM_2 NF-κB signature) across the different infection conditions. Higher expression values are displayed in yellow, while low expression values are shown in blue. **(g)** Umap plots showing expression values (in log-normalized counts) for the *Spag9* and *Slc15a3* genes in the integrated dataset. Higher expression values are displayed in yellow, while low expression values are shown in blue. **(h)** Dot plot showing scaled expression values among subsets of AMs for genes whose expression is associated with the AM_1 and AM_3 clusters in the integrated dataset. Higher expression levels are represented in yellow/green, while lower expression levels are shown in blue. The size of the dot corresponds to the percentage of cells that express each gene in each cluster. *n* = 5 for the infected population, *n* = 2 for the uninfected population. All the genes highlighted in the different figures show statistically significant greater expression in their clusters compared with cells in different clusters (FDR < 0.05; Wilcoxon rank-sum test; see Materials and methods).

AMs are a tissue-resident lung macrophage population (Hussell and Bell, 2014) and the first cell type to be infected by Mtb upon inhalation (Cohen et al., 2018; Srivastava et al., 2014). Population-based analysis indicates that this macrophage lineage is impaired in its T helper type 1 cell pro-inflammatory response and represents a niche for bacterial replication and dissemination in the lung (Cohen et al., 2018; Huang et al., 2018; Pisu et al., 2020a; Rothchild et al., 2019). Studies on AMs usually depend on the level of expression of SiglecF or CD11c surface markers to define the cell population. However, our current scRNA-seq analysis reveals two distinct sub-populations of AMs that mount pro-inflammatory responses and are associated with *hspx′*::GFP^high^ bacteria. First, a subpopulation in the AM_1 cluster (defined as AM_Pro-Infl; Fig. 3 a) in infected cells expresses high levels of *Nos2*, similar to IMs (Fig. 2 a). Trajectory and pseudotime analysis suggest that this population represents a cell fate originating from branching point 11 (Fig. 2 f). Comparison of the transcriptional profile of AM_Pro-Infl against the remaining cells in the AM_1 cluster revealed up-regulation of a pro-inflammatory gene signature shared by IMs (*Nos2*, *Clec4e*, *Saa3*, *Ccl5*, and *Ubd*), together with down-regulation of the *Marco* PRR, oxidative phosphorylation, and mitochondrial genes (Fig. 3 b, Fig. S4 b, and Table S8). Intriguingly, this pro-inflammatory AM population also up-regulates *H2-M2* (MHC-class IB), *Ubd*, and *Cxcl9* genes, implying an involvement in T cell infiltration and activation (Dangaj et al., 2019; House et al., 2020; Fig. 3 b). Pathway enrichment analysis confirms that glycolysis and nonsense-mediated mRNA decay are the main up-regulated gene signatures in AM_Pro-Infl (Fig. S4 a). ADT staining shows that this cluster maintains SiglecF expression, albeit at a lower level, while expression of CD63, CD11b, and CD38 is increased in both bystander and infected cells (Fig. 3, c and e).

The AM_2 cluster is interspersed within the IM_1 population in infected cells (Fig. 1 e), and comparison of its transcriptional profile against other AM subpopulations reveals up-regulation of a gene signature very similar and overlapping with IM subsets, including pro-inflammatory genes (*Nos2*, *Saa3*, *Clec4e*, and *Ccl5*) and IM_1 genes (Table S9, Table S10, Table S11, and Table S12). Similar to IM_1, 50% of the cells are associated with *hspx′*::GFP^high^ bacteria (Table 1), and intriguingly, this infected population stains negative for SiglecF (Fig. 3 c). Trajectory analysis indicates that cells in the AM_2 population either converge toward branching point 4 and IM_1 or represent a well-defined (pro-inflammatory) cell state (Fig. 2 f). This is indicated by the expression of *Zeb2* and *Cxcl2* marker genes, which are exclusive to the AM_2 population in uninfected mice, but become up-regulated by AM_2 and IM_1 bystander cells upon infection (Fig. 3 f and Fig. 2 d). Intriguingly, when looking at the infected cells, while *Zeb2* is expressed by both clusters (AM_2 and IM_1), *Cxcl2* is only expressed by IM_1, therefore indicating that subsets of cells in the AM_2 cluster may respond divergently to infection (Fig. 3 d). Recently, a comparable population of pro-inflammatory Cxcl2^+^ AMs has also been reported in response to pulmonary fungal infections in mice (Xu-Vanpala et al., 2020). Analysis of the full transcriptional profile of AM_2 in uninfected mice shows that these cells, although SiglecF^+^, overexpress NF-κB–mediated pro-inflammatory genes (*Malt1*, *Spag9*, *Cxcl2*, and *Slc15a3*; Shibolet et al., 2007; Song et al., 2018; Staal et al., 2011; Fig. 3, f and g), and are characterized by higher mitochondrial content (Fig. 5 d; discussed later) and up-regulated oxidative phosphorylation, which is comparable to the Cxcl2^+^ AMs described previously (Xu-Vanpala et al., 2020; Table S13, Table S14, and Table S15). Tracking the expression of this NF-κB gene signature across infection status (Fig. 3 f) reveals a pattern similar to *Zeb2* and *Cxcl2*, suggesting that upon infection with Mtb, a subpopulation of AM_2 cells segregates within the IM_1 cluster, while others mount a pro-inflammatory response that is similar to IM_3. Given the low level of expression of monocyte markers in the IM_1 cluster, it is likely that these cells constitute a mixed population generated from both IM_3 and AM_2 macrophages that have transitioned from a pro-inflammatory state toward a state of resolution. This is supported by the high expression of DNA regulators and chromatin remodeling genes within the AM_2 cluster of bystander cells (*Ankrd11*, *Baz1a*, *Cebpb*, *Bhlhe40*, and *Tra2b*; Table S16, Table S17, and Table S18).

It is now known that murine AMs are long-lived cells, capable of self-renewal and maintained independently of circulating monocytes (Guilliams et al., 2013; Hussell and Bell, 2014). In line with previous studies (Angelidis et al., 2019; Mould et al., 2019), our data indicate that the self-renewal capacity of AM populations is fulfilled by a specific cluster of replicating cells annotated as AM_4. Analysis of the transcriptional profile indicates that this is an actively proliferating population undergoing cell cycle, as confirmed by pathway analysis (data not shown) and expression of the canonical markers *Top2a* and *Mki67*, detailed later in mouse/human comparison (Fig. 5 b and Table S19).

Finally, AM_1 and AM_3 share a common gene signature typically associated with M2 polarization including expression of *Marco* and *Chil3* (*Ym1*) receptors (Fig. 3 h and Fig. S4 b) and a transcriptional profile demonstrated previously to be linked to the up-regulation of fatty acid metabolism in Mtb in AMs: *Mgll*, *Lpl*, *Trf*, *Tkt*, and *LipA* (Pisu et al., 2020a; Table S20 and Table S21). Trajectory analysis indicates that AM_1 represents a transitional phenotype, from where cells transition toward AM_3, AM_2, or AM_Pro-Infl states (Fig. 2 f). Overall, the four different AM subpopulations (AM_1, AM_2, AM_3, and AM_Pro-Infl) are distinguished by different degrees of expression of pro-inflammatory responses, with AM_2 and AM_Pro-Infl showing the highest expression of inflammatory genes and AM_3 the most anti-inflammatory.

### Transcriptional profiling of the bacterial cells reveals the link between Mtb phenotype and host cell responses

**Figure 4.**
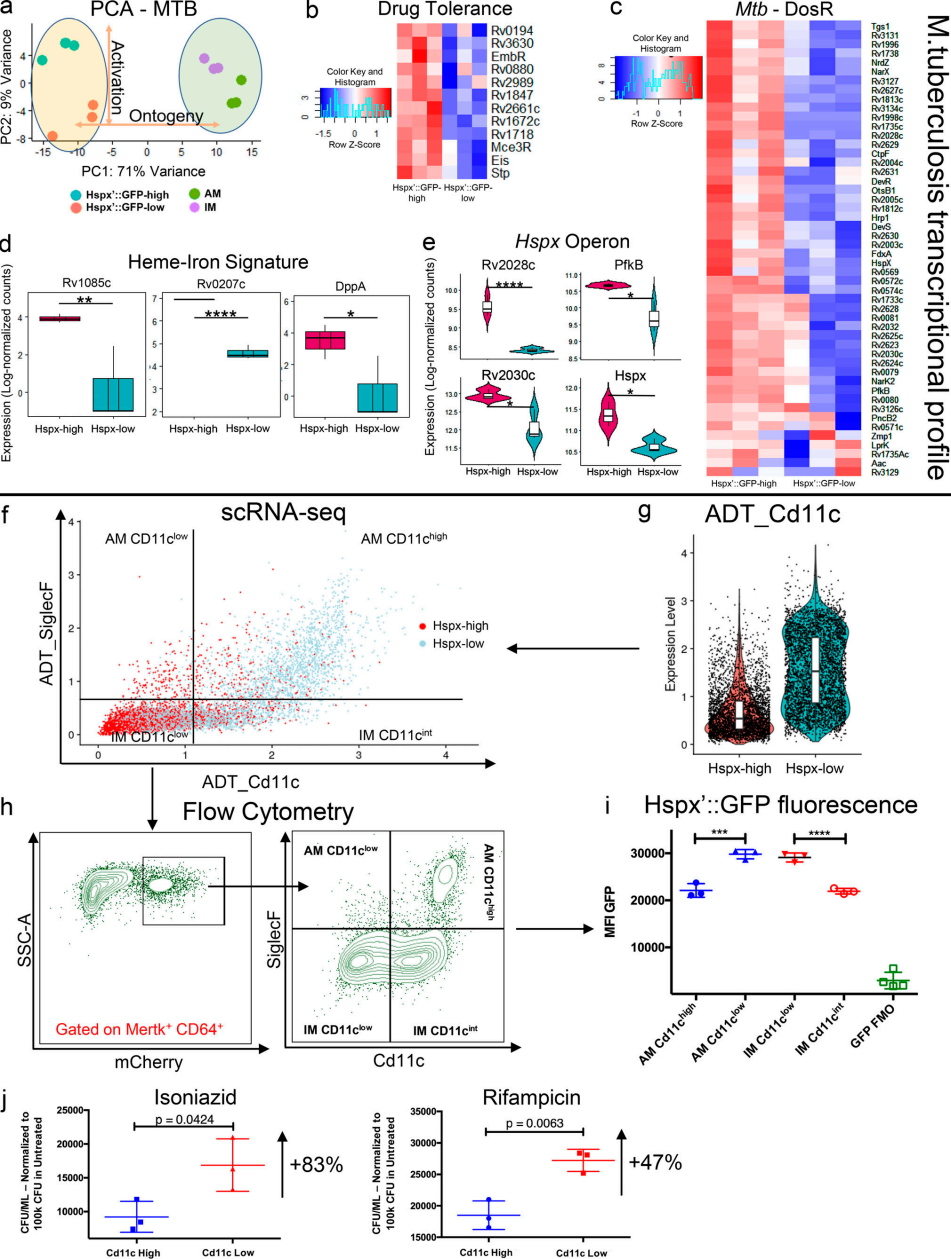
**Diverse bacterial transcriptomes and drug tolerance phenotypes are associated with expression of CD11c in the host cell. **Flow sorting of infected macrophages on the basis of Mtb GFP expression before dual RNA-seq analysis of the bacterial transcriptomes establishes links between bacterial stress, induction of drug tolerance, and the expression levels of the macrophage surface marker CD11c. **(a)** PCA of the Mtb transcriptome for the four different samples: *hspx′*::GFP^high^, *hspx′*::GFP^low^ (this manuscript), and Mtb in AM and IM (Pisu et al., 2020a). PCA reveals that both activation (*hspx′*::GFP^high^, *hspx′*::GFP^low^ samples) and ontogeny of the host immune cell (Mtb in AM, Mtb in IM) play a role in shaping bacterial responses during infection. **(b)** Heatmap showing relative expression levels for the Mtb drug tolerance gene signature. **(c)** Heatmap showing relative expression levels for the *DosR* regulon in Mtb. **(d)** Boxplots showing expression levels (in log-normalized counts) for the genes related to the heme-iron signature in Mtb. *hspx′*::GFP^high^ bacteria overexpress a set of genes involved in heme-iron uptake and catabolism in agreement with the finding that most *hspx′*::GFP^high^ bacteria are contained in macrophages that are heme-loaded. **(e)** Violin plots showing expression levels (in log-normalized counts) of the genes part of the *hspx* operon. **(f)** Scatter plots showing staining levels (in log-normalized values) for the surface markers CD11c and SiglecF in infected cells. Host cells containing *hspx′*::GFP^high^ bacteria are visualized in red, while cells containing *hspx′*::GFP^low^ Mtb are displayed in light blue. Four different populations of host cells (SiglecF^+^/CD11c^low^, SiglecF^+^/CD11c^high^, SiglecF^−^/CD11c^int^, and SiglecF^−^/CD11c^low^), each containing different proportions of associated *hspx′*::GFP^high^ bacteria, are identifiable. The data clearly show that expression of the surface marker CD11c is inversely correlated with the bacterial phenotype in both AMs and IMs. **(g)** Violin plot showing staining levels (in log-normalized values) for the surface marker CD11c in *hspx′*::GFP^high^- and *hspx′*::GFP^low^- infected host cells. **(h)** Flow cytometry analysis of infected macrophages (MerTK^+^ CD64^+^ mCherry^+^) at 3 w.p.i. from *hspx′*::GFP/*smyc′*::mCherry infected mice. The same populations we identified by scRNA-seq (f) are also visible by flow cytometry. SSC-A, side scatter area. **(i)** Flow cytometry analysis of the MFI for the GFP signal across the different subpopulations of infected macrophages (AM CD11c^high^, AM CD11c^low^, IM CD11c^low^, and IM CD11c^high^). **(j)** Quantification of the amount of Mtb that survived exposure to 1 μg/ml of either INH or RIF in CD11c^high^ and CD11c^low^ macrophages. Values have been normalized for 100,000 bacteria in the untreated group. An 83% and 47% increase in the number of bacteria that survived drug treatment in CD11c^low^ macrophages was observed for INH and RIF, respectively. *n* = 5 for the infected population, *n* = 2 for the uninfected population, *n* = 3 for the bacterial transcriptome. The statistical significance for the genes part of the bacterial transcriptome has been calculated using the Wald test as implemented in the DESeq2 package (FDR < 0.05; see Materials and methods; Love et al., 2014). The statistical significance is provided for the remaining plots part of the flow cytometry and drug tolerance experiments (*, p-adj. < 0.05; **, p-adj. < 0.01; ***, p-adj. < 0.001; and ****, p-adj. < 0.0001; one-way ANOVA with Tukey test and unpaired *t* test; see Materials and methods).

We recently developed a protocol to recover the transcriptional profile of Mtb from in vivo–infected host cells that revealed the differences in Mtb growth between the two main ontogenically defined macrophage populations: AMs and IMs (Pisu et al., 2020a). In the current study, to unravel the pathways used by Mtb to respond to the expression of potential anti-microbial responses by the host cell, we analyzed the transcriptional profiles associated with *hspx′*::GFP^high^ and *hspx′*::GFP^low^ bacteria recovered from the infected cells. Integration of the datasets (Mtb in AM, Mtb in IM, *hspx′*::GFP^high^, and *hspx′*::GFP^low^) reemphasizes that both macrophage ontogeny and the immune responsiveness of the host cell populations shape *Mtb* physiology during in vivo infection (Fig. 4 a). Comparison of the RNA-seq profiles from *hspx′*::GFP^high^ and *hspx′*::GFP^low^ bacteria showed that a total of 378 Mtb genes (286 up in *hspX′*::GFP^high^ and 92 up in *hspx′*::GFP^low^, false discovery rate [FDR] ≤ 0.05) were found to be differentially expressed (Table S22). *hspx′*::GFP^high^ bacteria up-regulate genes associated with phenotypic drug tolerance (*Rv1718*, *Rv1672c*, *Eis*, *Mce3R*, *Rv0880*, *Rv2661c*, *EmbR*, *Rv3630*, *Stp*, *Rv1847*, *Rv0194*, and *Rv2989*; Fig. 4 b); 34 (70%) genes of the *DosR* regulon (stress response; Fig. 4 c); the entire *Suf* operon (*Rv1460-Rv1466*), which encodes the primary Fe-S cluster biogenesis system (Fig. S4 d); the ergothioneine pathway (*EgtA-EgtD*) involved in detoxification of NO and ROS (Fig. S4 e); as well as the entire *Hspx* operon (*Rv2028c-Rv2031c*; Fig. 4 e). Most genes involved in iron acquisition and import are also up-regulated in *hspx′*::GFP^high^ bacteria (*IrtA*, *IrtB*, *MbtF*, *MbtD*, *MbtM*, *MbtN*, *MbtE*, *MbtB*, *MbtI*, *MbtC*, *MbtA*, *MbtH*, and *Rv1578c*), while *BfrB* (bacterioferritin, iron storage) is one of the top genes up-regulated by *hspx′*::GFP^low^ bacteria (Fig. S4 c). Intriguingly, the most highly expressed genes in the iron signature (*Rv0207c*, *Rv1085c*, and *DppA*) are associated with heme-iron uptake and catabolism (Fig. 4 d), in agreement with the finding that most *hspx′*::GFP^high^ bacteria are contained in macrophages that appear heme-loaded (IM_3). Among other genes highly expressed by *hspx′*::GFP^high^ bacteria are those involved in redox reactions (riboflavins and nicotinamide adenine dinocleotide synthesis), sulfatases, and more in general nitrogen detoxification and metabolism, suggesting that NO is the main source of stress for these bacteria (Table S22).

### Expression of the surface marker CD11c is inversely associated with drug-tolerant bacteria

To date, CD11c and SiglecF have been used as ontogenic markers to separate AMs from IMs. Recently, this notion has been challenged by the identification of a population of Mtb-infected monocyte-derived macrophages that are CD11c^high^ (Lee et al., 2020). Using ADT staining, we reclustered the infected cells based on the expression of surface markers rather than transcriptional profile. Intriguingly, we found that expression of CD11c in the host cell correlates inversely with the expression levels of *hspx′*::GFP (Fig. 4 g). By plotting CD11c against SiglecF expression, we found that *hspx′*::GFP^high^ bacteria are associated with CD11c^low^ cells in both AM and IM, suggesting that reduced expression of CD11c corresponds with increased M1 activation in macrophages (Fig. 4 f). Flow-cytometric analysis confirmed the scRNA-seq data as both CD11c^low^ AM and CD11c^low^ IM populations showed an average 35% increase in the median fluorescence intensity (MFI) of the GFP signal compared with CD11c^high^ AM and CD11c^int^ IM (Fig. 4, h and i).

Drug tolerance is defined as an increase in the survival times of bacterial cells toward a bactericidal drug without any change in the minimum inhibitory concentration and this phenomenon is frequently associated with a reduction of bacterial metabolism upon encountering specific environmental conditions (Boldrin et al., 2020). We had shown previously that the drug tolerance phenotype in Mtb can be induced through classical activation of macrophages in vitro and that nitrosative stress is the most potent single inducer of drug tolerance in Mtb in a murine infection in vivo (Liu et al., 2016). Because *hspx′*::GFP^high^ bacteria show overexpression of genes associated with stress and drug tolerance, we tested whether Mtb in CD11c^low^ macrophages exhibited increased survival in comparison with their counterparts in CD11c^high^ cells when exposed to the standard antitubercular drugs isoniazid (INH) and rifampicin (RIF). We infected mice for 3 wk using a constitutively expressed mCherry^+^ reporter strain, sorted CD11c^high^ and CD11c^low^ infected macrophages, and established these cells in culture (Fig. S4 f). We then treated half of the sorted population from each group with either 1 µg/ml INH or RIF or an equal amount of DMSO. We observed an average 83% increase in drug-tolerant bacteria for INH (CD11c^low^ mean ± SD, 16,870 ± 2,245; CD11c^high^ mean ± SD, 9,210 ± 1,323; *n* = 3) and an average 47% for RIF (CD11c^low^ mean ± SD, 27,225 ± 1,012; CD11c^high^ mean ± SD, 18,513 ± 1,313; *n* = 3) in CD11c^low^ versus CD11c^high^ macrophages (Fig. 4 j). These data confirm the Mtb expression profiles and indicate that the AM and IM populations consist of both permissive (CD11c^high^) and controller (CD11c^low^) sub-populations.

### Comparable macrophage subpopulations can be found in human airways

**Figure 5.**
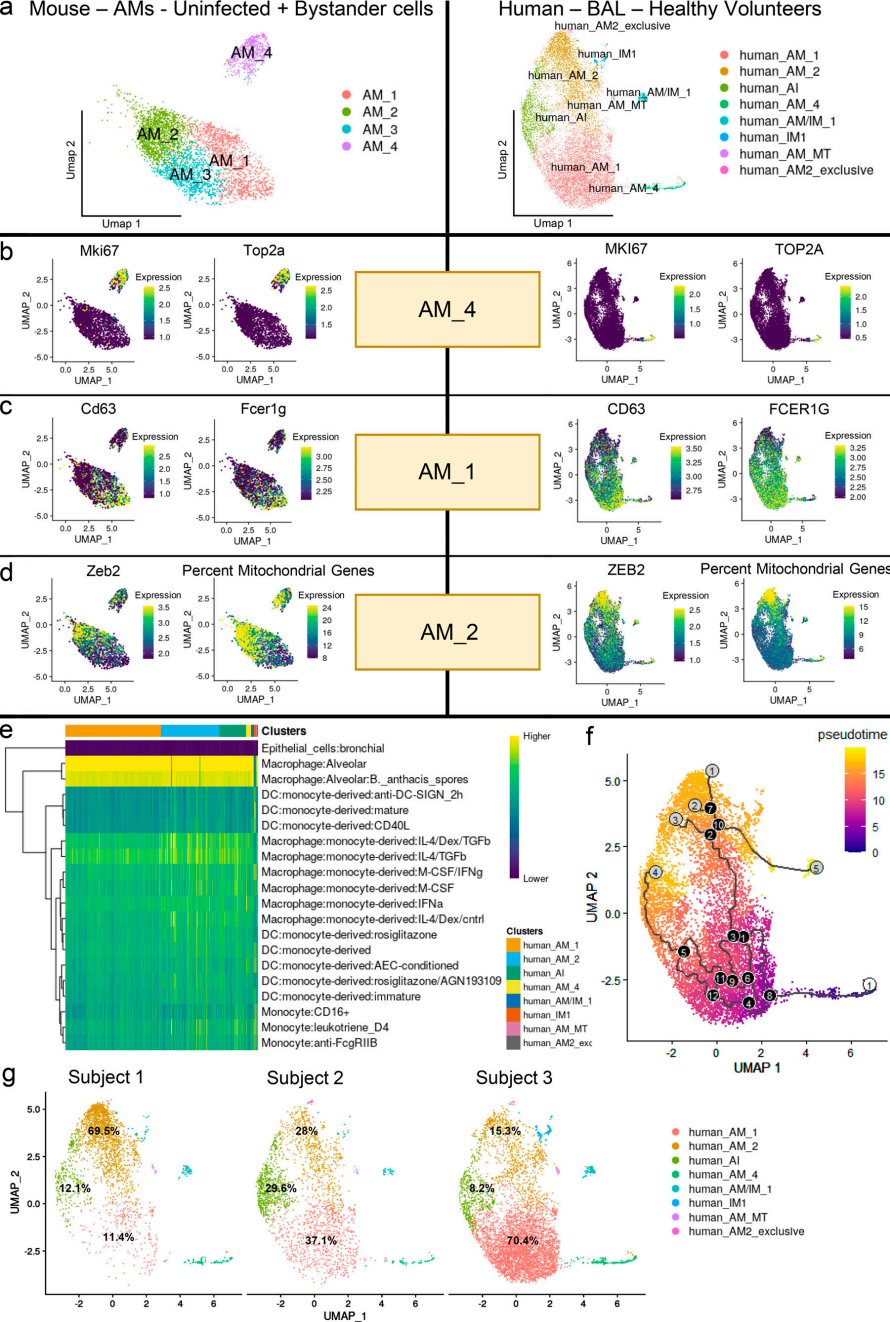
**Human–mouse comparison of the different AM subsets.** scRNA-seq of human lung macrophage populations from healthy volunteers identifies resident macrophage subsets that share transcriptional signatures of biological significance with murine macrophages. **(a)** Umap plots showing unbiased clustering of non–Mtb-infected AMs (uninfected + bystander cells) in mice and myeloid populations from BAL of healthy volunteers in humans. **(b)** Umap plots showing gene expression values (in log-normalized counts) for the marker genes (*Top2a* and *Mki67)* of the AM_4 population in both mice and humans. Higher expression values are displayed in yellow, while low expression values are shown in blue. **(c)** Umap plots showing gene expression values (in log-normalized counts) for the marker genes (*CD63* and *Fcer1g*) of the AM_1 population in both mice and humans. Higher expression values are displayed in yellow, while low expression values are shown in blue. **(d)** Umap plots showing gene expression values (in log-normalized counts) for the marker gene *Zeb2,* as well as the percentage of mitochondrial reads of the AM_2 population in both mice and humans. Higher expression values are displayed in yellow, while low expression values are shown in blue. **(e)** Heatmap showing the results of reference-based analysis for the human integrated dataset against the Human Primary Cell Atlas database. Higher similarity scores among the transcriptional profiles of query and reference datasets are showed in yellow. **(f)** Umap plot showing single-cell trajectory and pseudotime analysis of macrophage populations in the human integrated dataset. Gray circles represent cell fates, while black circles are defined as branching points through which cells transition in the trajectory. For pseudotime, cells farthest away from the root are colored in yellow, while cells close to the root are colored in blue. **(g)** Umap plot showing a split-view (by subject) of the unbiased clustering of the human dataset. As evidenced in the text, the proportions of the main AMs subsets vary considerably among different individuals. Values indicate the number of cells belonging to each cluster as a percentage of the total amount of cells recovered from each individual. *n* = 3 for the human BAL samples.

We wished to determine whether comparable macrophage subsets were present in human airways. We therefore recovered BAL samples from the lungs of three healthy BCG-vaccinated volunteers and performed scRNA-seq analysis and integrated the datasets as detailed. Reference-based analysis against the Human Primary Cell Atlas (Mabbott et al., 2013) confirmed that most BAL cells were AMs, as expected (Fig. 5 e). Significantly, we found that the major mouse lung AM subpopulations characterized in this study are also present in the lung of the healthy volunteers (Fig. 5 a): human AM_4, a subpopulation of highly proliferative, self-renewing cells, that express the same markers (*Top2a* and *Mki67*) as AM_4 in mice (Fig. 5 b and Table S23); a population, human AM_1, whose expression profile was similar to that of mouse AM Pro-Infl and AM_1, as evidenced by marker genes (*CD63*, *Fcer1g*, *Lgals3*, *Ndufa4*, *Fabp5*, and *S100a10*; Fig. 5 c and Table S24); and a population, human AM_2, that shares a transcriptional profile similar to mouse AM_2 and is characterized by expression of the marker gene *Zeb2* and increased oxidative phosphorylation and mitochondrial content (Fig. 5 d and Table S25).

Comparison of other lung macrophage populations identified a population of tissue resident cells, human IM_1, in close proximity to the human AM_2 cluster, whose transcriptional profile is similar to mouse IM_1, including expression of *Mafb*, *Zfp36*, *Klf6*, *Junb*, and *Zeb2* (Table S26); a very small population of AMs (as defined by reference-based analysis; Fig. 5 e), human AM/IM_1, in close proximity to the human AM_2 cluster, that also share a gene signature related to mouse IM_1, but that in comparison show higher expression of chemokine ligands (Table S27); a population of AMs, human alveolar/interstitial macrophages (AI), that has no obvious homologues and that expresses a mix of gene signatures that in mice are found in both AMs and IMs (Table S28); a small population of AMs, human AM_MT, that up-regulate expression of metallothionein genes, found only in humans (Table S29); and a very small subpopulation of human AM_2 macrophages with no clear homologues in mice (Table S30). Trajectory and pseudotime analysis also show a similar relationship between macrophage subpopulations to that observed in mice (Fig. 5 f).

Not surprisingly, given the known BAL cell heterogeneity between individuals in this cohort (Jambo et al., 2014; Mitsi et al., 2018; Mwale et al., 2018; Mwandumba et al., 2008), the proportions of these lung macrophage subpopulations vary between individuals, indicating that genetic variability and/or environmental factors likely influence the composition of the macrophage populations in the lung (Fig. 5 g). Our data show that these different macrophage subpopulations are associated with different bacterial fitness phenotypes in the mouse infection model, implying that a similar correlation would emerge in human tuberculosis infection.

### Epigenetic bias regulates macrophage response to mycobacterial stimulation

**Figure 6.**
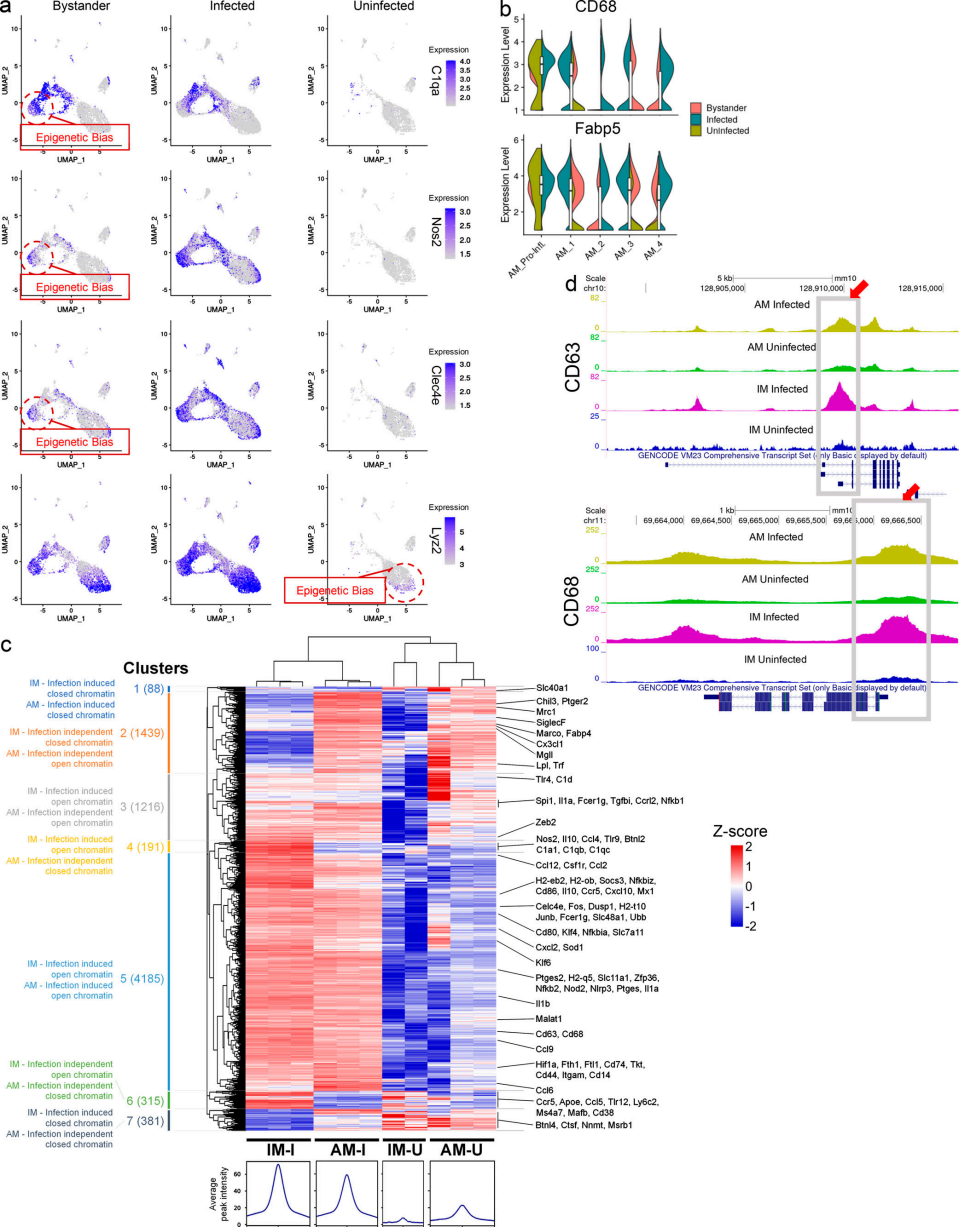
**Identification of open chromatin regions shared between the different subsets of macrophages in both Mtb and BCG infection. **Transcriptomic data from Mtb-infected mice, when compared with ATAC-seq data from mice following i.v. immunization with BCG, identify common patterns of epigenetic regulation shared by the IM and AM macrophage subsets in response to mycobacterial stimulation. **(a)** Umap plots (split-view) showing expression levels (in log-normalized counts) for the *C1qa*, *Nos2*, *Clec4e*, and *Lyz2* genes among the different infection conditions. As evidenced in the text, many of the pro-inflammatory genes are expressed even before infection (in both bystander and uninfected cells) by the same clusters that are associated with *hspx′*::GFP^high^ bacteria in the infected population. Cells with high levels of expression are highlighted in blue. **(b)** Violin plots showing expression levels (in log-normalized counts) for the *CD68* and *Fabp5* genes among different infection conditions. As noted in the text, both genes are uniquely overexpressed by the AM_Pro-Infl subpopulation even in the uninfected cells. **(c)** Heatmap showing relative differential peak intensity among BCG-infected and -uninfected AMs and IMs for the 7,815 chromatin promoter regions identified as DA by ATAC-seq. Seven different clusters were identified, the chromatin accessibility of which is changed depending on infection status and ontology of the host cell. **(d)** UCSC Genome Browser tracks for two of the genes of the AM_Pro-Infl signature, *CD63* and *CD68*. We detected the chromatin open state of the region for the active promoters in uninfected AMs, indicating that cells from this population are epigenetically constrained in their response to infection. *n* = 5 for the infected population, *n* = 2 for the uninfected population, *n* = 3 for the BCG-infected and uninfected ATAC-seq experiments. All the genes highlighted in a and b show statistically significant greater expression in their clusters compared with cells in different clusters (FDR < 0.05; Wilcoxon rank-sum test; see Materials and methods). Genes highlighted in panels c and d show statistically significant greater expression in each sample compared with all other samples (FDR < 0.05; quasi-likelihood F-test method; Lun et al., 2016; see Materials and methods). AM-I, AM infected; AM-U, AM uninfected; IM-I, IM infected; IM-U, IM uninfected.

The marked diversity across the transcriptional profiles of the mouse macrophage subpopulations in our scRNA-seq data, coupled with the observation that some expression patterns seem to precede infection, implies a degree of epigenetic programming, as recently reported for AMs (Xu-Vanpala et al., 2020). For example, we were surprised to observe that bystander IM_3 concurrently overexpressed both the complement gene signature (*C1qa*, *C1qb*, *C1qc*, and *Apoe*; Table S31) that characterizes infected IM_2 and, although at a lower level than the infected cells, the same gene signature associated with control of Mtb infection and *hspx′*::GFP^high^ bacteria (*Saa3*, *Clec4e*, *Ly6i*, *Nos2*, *Ccl5*, and *Hp*; Fig. 6 a and Table S32). In contrast, bystander IM_2s were characterized by a transcriptional signature that, other than the *C1qs* genes, did not overlap with their infected counterpart (*Ccl8*, *Lgmn*, *Timp2*, *Ms4a7*, and *CD83*; Table S33).

Looking at AM subpopulations, we observed similar trends. Focusing on the AM Pro-Infl cluster, we identified a conserved gene signature expressed by this subpopulation irrespective of infection status (*Ftl1*, *Fth1*, *Fabp5*, *Lyz2*, *CD68*, and *Cd63*; Fig. 6, a and b; and Fig. S2 e), suggesting this subpopulation of pro-inflammatory AMs is already present in the lungs of uninfected mice (and humans, as detailed previously). We therefore hypothesized that the different responses to Mtb infection that we observed among the infected cells may be a direct consequence of the preexisting chromatin organization (epigenetic bias) among the different populations.

*M. bovis* BCG is known to induce Mtb-comparable innate immune activation in myeloid cells (Geisel et al., 2005; Kaufmann et al., 2018; Khan et al., 2020; Rhoades et al., 2005), and there is increased interest in the efficacy of BCG as an inducer of protective immunity against tuberculosis, as recent data showed that i.v. vaccination with BCG prevents or substantially limits Mtb infection in highly susceptible rhesus macaques (Darrah et al., 2020; Nemes et al., 2018). Intravenous infection with BCG induces a reprogramming in macrophage responses known as “trained immunity” that is mediated, at least in part, through epigenetic control of transcriptional activity (Khader et al., 2019). We therefore used live BCG infection to assess the changes in chromatin organization of AMs and IMs and determine if i.v. BCG vaccination generates similar immunological responses to those observed with Mtb infection in mice.

**Figure S5.**
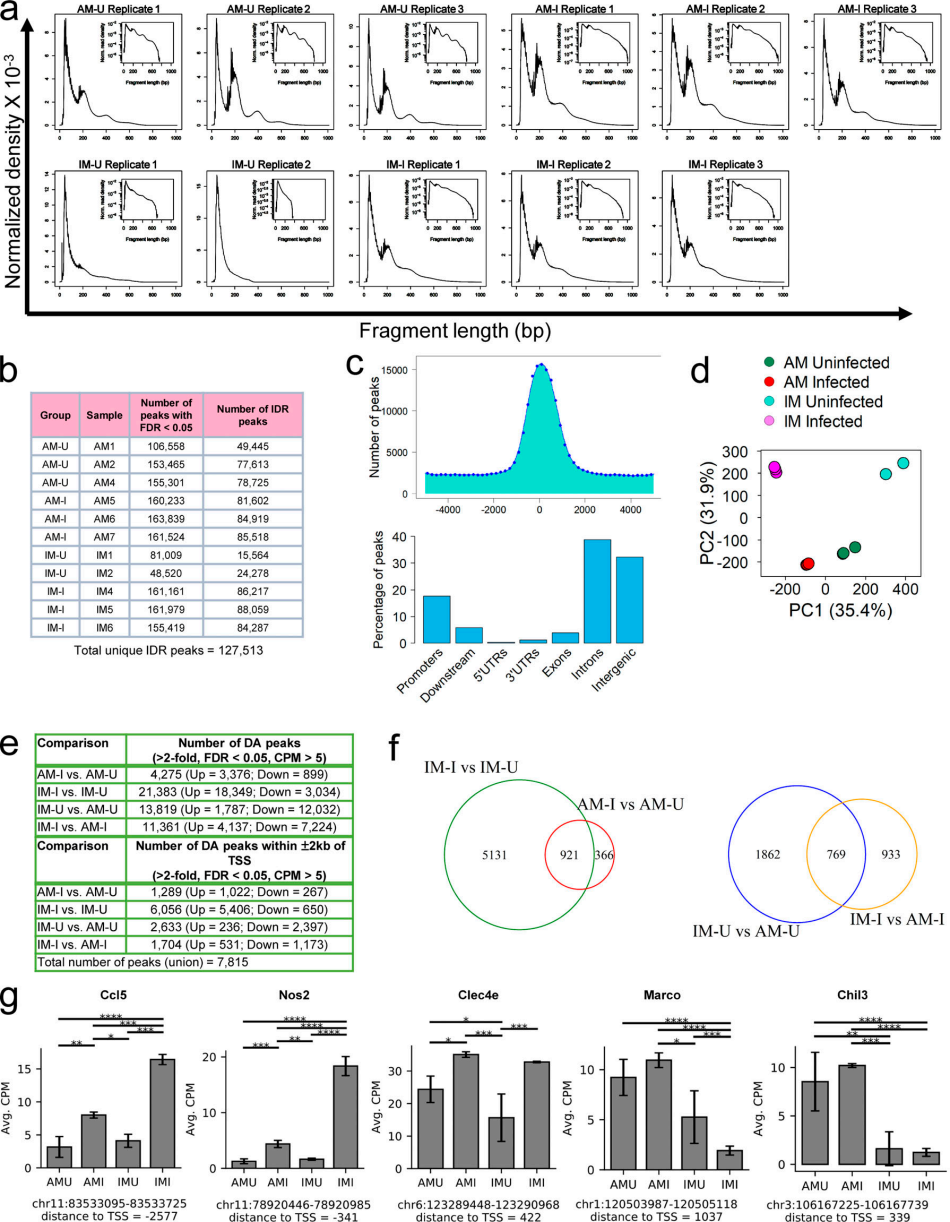
**ATAC-seq QC plots and DA peak average CPM mapped reads at the promoter regions for genes that characterize the major macrophage subpopulations. (a)** Histograms showing the distribution of fragment lengths for each of the replicates. **(b)** Table showing the number of called peaks (FDR < 0.05) and IDR peaks (IDR < 0.05) for each of the replicates. **(c)** Distribution of called peaks based on the distance from the TSS (top) and percentages of called peaks among different genetic regions (bottom). **(d)** PCA plot of the ATAC-seq peak tag counts data, segregated samples by infection status (PC1) and cell type (PC2). **(e)** Tables showing the total number of DA peaks (FC more than twofold; FDR < 0.05; CPM > 5; top), and the number of DA peaks (FC more than twofold; FDR < 0.05; CPM > 5) in the promoter regions (± 2 kb from TSS) across different comparisons (bottom). **(f)** Venn diagrams showing the number of DA peaks in the promoter regions when comparing cell types (left) or infection status (right). **(g)** Bar plots showing the average CPM at the promoter region for the *Nos2*, *Ccl5*, *Clec4e*, *Marco*, and *Chil3* genes. *n* = 3 for the BCG-infected and uninfected ATAC-seq experiments. Unless otherwise specified, the statistical significance is provided when appropriate for each plot (*, p-adj. < 0.05; **, p-adj. < 0.01; ***, p-adj. < 0.001; and ****, p-adj. < 0.0001; quasi-likelihood F-test method; Lun et al., 2016; see Materials and methods). Avg., average; UTR, untranslated region; AM-I, AM infected; AM-U, AM uninfected; IM-I, IM infected; IM-U, IM uninfected.

We used ATAC-seq to identify the regions of the genome associated with open chromatin peaks in the two ontologically distinct macrophage subsets, in both BCG-infected and -uninfected mouse lungs (Fig. S5, a–c). An unbiased genome-wide comparison of ATAC-seq peak tag counts data segregated samples by infection status (principal component [PC] 1, 35.4%) and cell type (PC2, 31.9%; Fig. S5 d). Pairwise comparison of ATAC-seq peak tag counts revealed several thousand genomic sites with differentially (fold change [FC] > 2, FDR < 0.05, counts per million [CPM] > 5) opened chromatin between AM and IM in both BCG-infected and -uninfected mouse lungs (Fig. S5 e, top). When looked within ±2 Kb of the transcriptional start site (TSS), a union of 7,815 differentially accessible (DA) peaks was observed (Fig. S5 e, bottom; and Table S34). Most of the DA peaks, i.e., 6,056, were observed in infected IMs over uninfected IMs (Fig. S5 e, bottom). Among these, 921 DA peaks were shared with the comparison of infected AMs versus uninfected AMs (Fig. S5 f), indicating a common response to infection in both macrophage subsets.

Clustering of uninfected and infected IMs and AMs on these 7,815 DA peaks resulted in the identification of seven specific clusters (Fig. 6 c, top), the chromatin accessibility of which was changed drastically upon infection (Fig. 6 c, bottom). We noticed that chromatin-accessible patterns overall coincide with the scRNA-seq datasets. In particular, DA peaks in cluster 2 (infection-independent, open chromatin in AM) were associated with the gene signature that, in the scRNA-seq dataset, characterizes AMs across all infection conditions, while cluster 3 (infection-induced in IM) correlated to the gene signature associated with IM_1. DA peaks in cluster 4 (infection-induced in IM) define genes up-regulated by IMs, while DA peaks in cluster 5 (infection-induced in both AM and IM) were associated with a gene signature up-regulated by both IMs and AMs in infected cells in the scRNA-seq dataset. Finally, DA peaks in cluster 6 (infection-independent in IM) were associated with genes that define the IM_2 and IM_3 subsets (Table S2, Table S3, Table S4, and Table S34). In detail, when looking at IMs, we observed a highly open chromatin pattern for all complement genes (*C1qa*, *C1qb*, and *C1qc*) in both uninfected and BCG-infected IMs relative to their AM counterpart, (cluster 4; Fig. 6 c). IMs also showed an open chromatin pattern among pro-inflammatory genes, such as *Ccl5*, *Ccl4*, and *Nos2* from infected mice (Fig. S5 g). AMs exhibited specific accessibility in M2 receptors such as *Marco* and *Chil3* (cluster 2; Fig. 6 c and Fig. S5 g) and metabolic genes such as *Mgll*, *Lpl*, *Trf*, and *Tkt* (cluster 2; Fig. 6 c). The chromatin regions of these genes in IMs were closed (Fig. 6 c and Fig. S5 g). Chromatin accessibility for pro-inflammatory and infection-associated genes such as *Il1b*, *Il6*, *Nlrp3*, *Itgam* (*Cd11b*), *CD80*, *CD14*, *Fos*, and *Jun* were also increased in infected AMs and IMs (cluster 5; Fig. 6 c). Intriguingly, the gene signature that characterizes the AM_Pro-Infl population (Fig. 5 c and Fig. 6, b and d), which was also present in the lung of uninfected mice, had open chromatin in uninfected AMs relative to uninfected IMs and was also opened in infected AMs and IMs (Fig. 6 d), in support of the contention that different macrophage subpopulations are epigenetically constrained in their response to infection. Overall, our analysis suggests that the preexisting chromatin organization of the different subsets of macrophages is, at least in part, involved in driving the resulting gene expression profiles upon infection. Because the ATAC-seq data generated from a different infection model (BCG) show a high degree of correlation with the gene expression patterns observed in Mtb-infected lungs, it is likely that the divergent host cell responses to either BCG or Mtb infection are a combination of epigenetic imprinting that exists before infection, in addition to the subsequent responses to the evolving cytokine environment.

## Discussion

In this study, we have used scRNA-seq to characterize the heterogeneity among the macrophage cell lineages present in the Mtb-infected and uninfected mouse lung at a single time point after challenge. The study is not intended to serve as a model for disease progression but to generate an appreciation of the functional heterogeneity of the host macrophage populations with respect to their ability to control or promote bacterial growth. Recent work has shown that the myeloid cell populations in tissues such as the lung are heterogeneous, with respect to both origin and their responsiveness to insult, infection, and immune stimulation (Huang et al., 2018; Mould et al., 2017). Where this current study is novel and extends our understanding is through the use of fluorescent fitness reporter strains to phenotypically type the infected cells before sorting and scRNA-seq. This experimental and analytical pipeline facilitates identification of controller and permissive host cell phenotypes, which in turn enables the linkage of different bacterial phenotypes to transcriptionally distinct AM and IM subpopulations. This functional linkage between host transcriptome and bacterial fitness provides a deeper understanding of the roles played by the divergent responses of macrophage subsets in shaping the course of Mtb infection.

Our previous work indicated that the AMs were more permissive to bacterial growth than the IMs, and this is true on the population level. However, what is clear from Fig. 1 b and Fig. 2 a, where the IM and AM cell populations are overlaid with the bacterial stress response readout (*hspx′*::GFP), is that both the IM and AM lineages contain permissive and controller sub-populations. The *hspx′*::GFP reporter had been shown previously to be primarily responsive to NO, and the *hspx′*::GFP^high^ Mtb-infected host cells closely matched *Nos2*^*+*^ expression. Both the IM and AM lineages contain four discernable subpopulations that can be defined on the basis of their transcriptional profiles. The IM_1 subpopulation appears to be preexisting in uninfected mouse lung tissue and expresses *Zeb2*, which has been correlated with maintenance in the tissues. In infection, these cells exhibit an 1L-17a–mediated profile and up-regulate genes associated with resistance to tuberculosis progression. The IM_3 subpopulation is relatively homogeneous; appears stressed, possibly by iron overload; and up-regulates expression of the Nos2 protein. The IM_2 population represents a more heterogeneous cluster exhibiting a Nrf2 signature, which has been associated with a reduced inflammatory response (Qian et al., 2016; Rothchild et al., 2019).

While overall the AM populations appear less diverse, they too consist of four discernible subpopulations. The AM_1 population also contains a subset, designated AM_Pro-infl, that expressed high levels of *Nos2*, and showed up-regulation of proinflammatory genes and transcripts associated with glycolysis. A similar population of inflammatory AMs was also present in the AM_2 cluster and seems closely related to IM with respect to transcriptional profile. Both AM_1 and AM_3 populations shared a common signature usually associated with M2, or alternatively activated macrophages, and these are the cells linked to Mtb with low induction of the *hspx′*::GFP reporter (Fig. 2 a). Finally, the transcriptome of the AM_4 cells revealed a phenotype of cell division suggesting that this subset is responsible for the maintenance and replenishment of the AM population. Pseudotime analysis of the macrophage populations suggests the sequence through which the IM and AM cells may transition between subsets or states (Fig. 2 f).

IMs are predominantly controller cells in phenotype; however, the IM_2 and IM_1 subpopulations do contain subsets of cells that, through low induction of *hspx′*::GFP expression, appear to be permissive to bacterial growth. In a previous study, we demonstrated that host cell activation correlated with induction of drug tolerance (Liu et al., 2016; Sukumar et al., 2014), indicating that immune containment runs contrary to drug susceptibility. Consistent with this observation, we found that expression of CD11c correlated inversely with both bacterial stress (*hspx′*::GFP) and drug tolerance, which indicates that CD11c^+^ IMs are permissive for Mtb growth. The identification of a CD11c^+^, Mtb-infected IM population both in our study and that of Lee et al. (2020) raises the possibility that recruited, monocyte-derived macrophages can also give rise to a subpopulation of permissive host phagocytes capable of supporting bacterial expansion and disease progression.

Preliminary analysis of cells recovered by BAL from healthy human volunteers identified lung macrophage subpopulations in common between both species. The relative proportion of human AM subpopulations varied considerably between volunteers, which is consistent with donor variability and the known impact of environmental factors on the cells in the human airway (Mitsi et al., 2018; Mwale et al., 2018). However, the significance of the presence of these macrophage subpopulations before infection with Mtb suggests that the cell subpopulations already exist in the lung and may respond in a manner similar to that characterized in the mouse. Clearly, such a hypothesis requires further study in individuals with active tuberculosis to examine the links between macrophage identity and function and disease status. It will be interesting to determine whether bacterial stress reporters, such as hspx′::GFP, exhibit a response comparable to that observed in the mouse lung, in human AM/IMs following ex vivo challenge.

For many years, the macrophage field has been shaped by the M1/M2 polarization paradigm that macrophages exist in a nonprogrammed, neutral state until cytokine exposure determines their functional phenotype as either type 1 (IFN-γ and TNF-α) or type 2 (IL-4 and IL-13) activated macrophages, as discussed (Murray, 2017). While investigators have frequently noted that this paradigm fails to adequately capture phagocyte heterogeneity observed in vivo, the broad implications of this model have persisted, and the functional significance of macrophage heterogeneity has remained underappreciated (Russell et al., 2019). The current study provides additional insight at the single-cell level, linked to functional characterization, that demonstrates that the preexisting identity of the macrophage subpopulation exerts an extremely powerful influence over the response to infection. Previous work examining the impact of BCG on the trained immune response of macrophages in vivo indicated that much of that response came as a consequence of epigenetic regulation (Kaufmann et al., 2018). Here, we used ATAC-seq to study the chromosomal organization of the main macrophage lung subsets (AMs and IMs) before and after BCG infection. The degree of congruence between the epigenetic modifications induced by BCG, and the transcriptional response of the IM and AM populations in the mouse Mtb challenge model, were marked for several well-characterized, epigenetically regulated gene signatures. The results indicated that mycobacterial insult, whether through experimental vaccination or infection, will result in the induction of similar functional phenotypes in the host macrophage phagocytes. This implies that future vaccination strategies need to look at the epigenetic reprogramming of the innate immune response (Khader et al., 2019; Khan et al., 2020), in addition to consideration of the effectiveness of any T cell stimulation.

The current study builds on previous work to integrate single cell transcriptional profiling with functional phenotypes relevant to intracellular Mtb infection. Marrying the single cell heterogeneity with bacterial fitness has enabled us to identify controller and permissive host cell subsets in both the AM and IM macrophage lineages. While much of the existing literature on immune-mediated control deals with microbicidal responses, we believe that it is control of growth that is likely to be the dominant pathway for disease limitation in tuberculosis, where eradication of infection appears rare (Getahun et al., 2015). As the magnitude of the infecting bacterial population is impacted by the relative dimensions of the host macrophage subpopulations, it is critical that we use the increased resolution of scRNA-seq, in tandem with functional readouts, to define pathways of bacterial growth control within the macrophage subsets, and target modulation of these pathways to increase vaccine efficacy.

## Materials and methods

### Mtb and BCG strains

*Mycobacterium tuberculosis* Erdman (American Type Culture Collection 35801) was the parental strain used for all experiments. The fluorescent reporters *smyc′*::mCherry, *hspx′*::GFP/*smyc′*::mCherry, and *hsp60′*::GFP were previously described (Abramovitch et al., 2011; Dhandayuthapani et al., 1995; Sukumar et al., 2014; Tan et al., 2013). Bacteria were grown at 37°C to mid-log phase in MiddleBrook 7H9 broth supplemented with 10% oleic acid/albumin/dextrose/catalase (OADC Enrichment; Becton, Dickinson and Company), 0.2% glycerol, and 0.05% tyloxapol (Sigma-Aldrich). BCG (Pasteur) was grown as described above. Hygromycin B (50 mg/ml) was used as a selection marker for the fluorescent strains. For mice infection, aliquots were frozen in 10% glycerol, titered, and stored at −80°C until use.

### Mice

C57BL/6J WT mice were purchased from The Jackson Laboratory. The mice used in this study were 6–8 wk old. All mice were maintained in a specific pathogen–free animal biosafety level 3 facility at Cornell University. Animal care was in accordance with the guidelines of the Association for Assessment and Accreditation of Laboratory Animal Care. All animal procedures were approved by the Institutional Animal Care and Use Committee of Cornell University.

### Human subjects

BAL samples from human volunteers were obtained at the Queen Elizabeth Central Hospital, a large teaching hospital in Blantyre, Malawi. Participants were recruited from the hospital’s voluntary counseling and testing clinic. They were healthy, HIV-1–uninfected adults aged ≥18 yr, with no clinical, laboratory, or chest radiographical evidence of active pulmonary tuberculosis or other respiratory disease, willing to undergo bronchoscopy and BAL specifically for research purposes. HIV testing was performed on whole blood using two commercial point-of-care rapid HIV test kits, the Determine HIV 1/2 kit (Abbott) and the Uni-Gold HIV kit (Trinity Biotech). A participant was considered HIV-uninfected if the test was negative by both kits. If Determine and Uni-Gold results were discordant, a third rapid test using the Bioline HIV 1/2 kit (Standard Diagnostics Inc.) was performed to resolve the discordance. All the test kits used had reported sensitivity and specificity of 99–100% (van den Berk et al., 2003; Vijayakumar et al., 2005). The study received ethical approval from the research ethics committees of the University of Malawi College of Medicine (research protocol P.02-18-2356) and the Liverpool School of Tropical Medicine (research protocol 17–032). All study participants provided written informed consent.

### Mice infection and lung cells isolation

For Mtb, mice were anesthetized and inoculated intranasally with ∼1,500 CFU of one of the Erdman strains (*smyc′*::mCherry, *hspx′*::GFP/*smyc′*::mCherry, and *hsp60′*::GFP) resuspended in 30 μl of PBS containing 0.05% Tween 80. Inoculum dosage was confirmed by plating different dilutions of the bacterial stocks in 7H10 agar plates supplemented with OADC Enrichment, glycerol, and hygromycin B. Plates were incubated at 37°C and colonies enumerated 3 wk after. 3 wk post-infection (w.p.i.), mice were sacrificed, and the lungs were aseptically removed and placed in PBS containing 5% FBS. To minimize unwanted changes in the gene expression profile of both host and bacteria, samples were kept on ice and immediately processed using a GentleMACS tissue dissociator (Miltenyi Biotec; Pisu et al., 2020b). The dissociated lung material was then passed through a 70-µM cell strainer, and red blood cells were lysed with ammonium-chloride-potassium lysis buffer (Lonza).

For BCG, mice were intravenously infected with 10^6^ BCG (Pasteur) bacilli. Lung cell suspensions were obtained as detailed above.

### Sorting of the different murine populations for scRNA-seq analysis

#### Infected populations

Single-cell suspensions from infected mice (*n* = 5; three batches; total 15 mice) were washed in PBS plus 5% FBS, resuspended in sorting buffer (PBS, 5% FBS, 5 mM EDTA, and 25 mM Hepes), passed through a 40-µM cell strainer, and sorted according to the gating strategy depicted in Fig. S1 a. Samples were maintained at 4°C during sorting and collected directly into Cell Staining Buffer (BioLegend). Mice infected with either *smyc′*::mCherry or *hsp60′*::GFP were used as a control to define the sorting gates for the *hspx′*::GFP/*smyc′*::mCherry-infected cells (Fig. S1 a). In our datasets, we recovered a small number of infected neutrophils (3.5% of the myeloid cells), which we believe under-represents infected neutrophils (Huang et al., 2018). This is consistent with previous 10X Genomics studies (Esaulova et al., 2021; 10X Genomics, 2018).

#### Bystander populations

Single-cell suspensions from mice infected with *smyc′*::mCherry (*n* = 3; two batches; total six mice) were incubated for 20 min in the dark with fluorophore-conjugated antibodies against CD45 (104; BD). Stained samples were washed twice in PBS, resuspended in sorting buffer, passed through a 40-µM strainer, and sorted according to the gating strategy depicted in Fig. S1 b. Samples were maintained at 4°C during sorting and collected directly into Cell Staining Buffer (BioLegend).

#### Uninfected populations

Single-cell suspensions from uninfected healthy mice (*n* = 3; two batches; total six mice) were incubated for 20 min in the dark with fluorophore-conjugated antibodies against CD45 (104; BD). Stained samples were washed twice in PBS, resuspended in sorting buffer, passed through a 40-µM strainer, and sorted according to the gating strategy depicted in Fig. S1 c. Samples were maintained at 4°C during sorting and collected directly into Cell Staining Buffer (BioLegend). Unstained uninfected mice were used to define the sorting gate for the CD45 signal (Fig. S1 c).

### Sorting of murine AM and IM populations for ATAC-seq

AM and IM were sorted from lung single-cell suspensions prepared as described above using a BD FACSAria sorter. Cells were sorted in PBS containing 0.04% BSA and processed for ATAC-seq library preparation immediately.

### Sorting of host cells for recovering of the bacterial-associated transcriptomes

To recover the bacterial transcriptome associated with *hspx′*::GFP^high^ and *hspx′*::GFP^low^ bacteria, experimental samples comparable to the ones used for scRNA-seq have been processed using the Dual RNA-seq protocol developed previously (Pisu et al., 2020a). Briefly, single-cell suspensions from mice infected with *hspx′*::GFP/*smyc′*::mCherry Erdman (*n* = 3; three batches; total of nine mice) have been prepared as described above. The two different populations of infected host cells (*hspx′*::GFP^high^ and *hspx′*::GFP^low^) have been sorted (Fig. S1 a) and collected directly in Trizol, as previously described (Pisu et al., 2020a).

### BAL collection from human volunteers

Bronchoscopy and BAL were performed as described previously (Jambo et al., 2011; Mwandumba et al., 2004). A cell suspension containing 5 × 10^6^ BAL cells was centrifuged at 500 *g* for 8 min at 4°C, the supernatant was discarded, and the cells were resuspended in 1 ml of chilled 90% methanol (Merck Life Science). Fixed samples were stored at −80°C before shipping to Cornell University for scRNA-seq.

### scRNA-seq libraries preparation and sequencing

Sorted murine populations were stained with HTO and ADT antibodies (BioLegend) following published protocols (Stoeckius et al., 2017), with slight modifications. Briefly, sorted cells were spun down at 500 *g* for 5 min, resuspended in 50 µl of cell staining buffer containing 0.25 µg of TruStain FcX PLUS (anti-mouse CD16/32 blocking reagent; BioLegend), and incubated for 10 min at 4°C. 50 µl of an ADT plus HTO antibody cocktail mix was then added to the samples and incubated for another 30 min at 4°C. After incubation, samples were washed 2× in cell staining buffer, and differentially tagged samples (*hspx′*::GFP^high^/ *hspx′*::GFP^low^ and bystander/uninfected) were mixed and resuspended in Dulbecco’s PBS 1×. Ice-cold methanol was then added drop by drop to a final concentration of 90% (vol/vol). Fixed samples were stored at −20°C overnight. After fixation, samples were taken out of the BSL3 facility, equilibrated on ice for 15 min, and washed twice with rehydration buffer (1× Dulbecco’s PBS containing 1.0% BSA [Thermo Fisher Scientific] and 0.5 U/µl RNase Inhibitor [Sigma-Aldrich]), and the number of recovered cells was quantified before loading into the 10× chip. For the mRNA library preparation, we followed the commercially available 10× protocol (CG000206 Rev D), with a slight modification in step 2.2 and the addition of 1 μl of ADT and HTO additive primers (0.2 μM stock) as previously described (Stoeckius et al., 2017).

Fixed BAL samples were equilibrated on ice for 15 min, washed twice in rehydration buffer to remove residual methanol, stained with different HTO antibodies for 30 min at 4°C (as described above), and washed twice in cell staining buffer plus 0.5 U/μl of RNase inhibitor (Sigma-Aldrich). Subsequently, the number of cells recovered from each sample has been quantified, and different samples were mixed together in a 1:1 ratio and multiplexed into the same 10× run. Libraries were generated as detailed above.

For both murine and human samples, HTO and ADT libraries were generated following BioLegend commercially available protocols. mRNA, HTO, and ADT libraries were assessed for quality control (QC) using a Fragment Analyzer (Agilent); quantified by digital PCR (QX200; Bio-Rad); pooled together using the following proportions: murine (85% mRNA, 10% ADT, and 5% HTO) and human (90% mRNA and 10% HTO); and sequenced on a NextSeq500 (Illumina) using the 75-bp NextSeq kit with the following cycles: read 1 (28 cycles), i7 index (8 cycles), and read 2 (55 cycles), at a depth of >50,000 reads/cell.

### Antibody list for scRNA-seq

The following TotalSeq (BioLegend) murine antibodies were included in the antibody cocktail mix at a concentration of 0.5 μg/sample: SiglecF (custom-made; clone S17007L), CD64 (cat. # 139325), Ly6G (cat. # 127655), CD11c (cat. # 117355), CD14 (cat. # 123333), CCR5 (cat. # 107019), Ly6G-Ly6C (cat. # 108459), CD63 (cat. # 143915), F4/80 (cat. # 123153), CD38 (cat. # 102733), TLR4 (cat. # 117614), CD11b (cat. # 101265), CD16/32 (cat. # 101343), CD86 (cat. # 105047), CD1d (cat. # 123529), CD3 (cat. # 100251), CD4 (cat. # 100569), and CD8a (cat. # 100773). For hashing, we used the following antibodies from BioLegend: Hashtag 1 murine (cat. # 155801), Hashtag 2 murine (cat. # 155803), Hashtag 1 human (cat. # 394601), and Hashtag 2 human (cat. # 394603).

### ATAC-seq libraries preparation and sequencing

FACS-sorted cells were spun down at 500 *g* for 5 min, followed by a wash using 50 ml of cold 1× PBS and centrifugation at 500 *g* for 5 min. Cells were lysed using cold lysis buffer (10 mM Tris-HCl, pH 7.4, 10 mM NaCl, 3 mM MgCl_2_, and 0.1% IGEPAL CA-630), and immediately after lysis, nuclei were spun at 500 *g* for 10 min. Following the nuclei prep, the pellet was resuspended in the transposase reaction mix (25 µl 2× Tagment DNA buffer, 2.5 µl transposase [Illumina], and 22.5 µl nuclease-free water). The transposition reaction was performed for 30 min at 37°C. Directly following transposition, the sample was purified using a Qiagen MinElute kit. Then, we amplified library fragments using 1× NEBNext PCR Master Mix and 1.25 µM of custom Nextera PCR primers using the following PCR conditions: 72°C for 5 min; 98°C for 30 s; and thermocycling at 98°C for 10 s, 63°C for 30 s, and 72°C for 1 min. The libraries were purified using a Qiagen PCR cleanup kit in 20 µl. Libraries were amplified for a total of 10–13 cycles and were subjected to high-throughput sequencing using the Illumina HiSeq 2500 Sequencer (paired-end).

### RNA extraction and Mtb libraries preparation and sequencing

RNA extraction for Mtb transcripts has been performed as we previously reported (Pisu et al., 2020a). Ribosomal RNA (rRNA) depletion has been performed using a 1:1 mix of depletion solutions from the Ribo-Zero H/M/R and Ribo-Zero Gram+ kits (Illumina) and a modified protocol previously described (Pisu et al., 2020b). The rRNA-depleted samples were purified by ethanol precipitation. Sequencing libraries were generated using the NEBNext Ultra II Directional RNA Library Prep Kit for Illumina (New England Biolabs). Libraries were sequenced on a Novaseq 6000 S4 (Illumina) in multiple rounds until the desired sequencing depth for bacterial reads was reached (target 1 million reads, 2× nt paired-end reads).

### Flow cytometry analysis

Lung cell suspensions were counted and incubated for 30 min in the dark at room temperature with fluorophore-conjugated antibodies, washed twice with PBS 1×, and fixed in 4% paraformaldehyde. Antibody panels and Fluorescence Minus One controls were generated as appropriate. For this study, we used fluorochrome-conjugated mAbs specific to mouse SiglecF (E50-2440; Becton Dickinson), Cd11c (N418; BioLegend), CD64 (X54-5/7.1; BioLegend), MerTK (2B10C42; BioLegend), and CD45 (104; Becton Dickinson), along with the following reporter strains: *smyc′*::mCherry (mCherry), *hsp60′*::GFP (GFP), and *hspx′*::GFP/*smyc′*::mCherry. Cells were analyzed with an Attune NxT flow cytometer (Thermo Fisher Scientific). Data were analyzed using FlowJo software (version 10.7; BD).

### Measurement of drug tolerance of Mtb in murine macrophages

To assess the drug tolerance phenotype of Mtb in macrophages with different levels of expression of CD11c, mice were infected intranasally with ∼1.5 × 10^3^ CFUs of *smyc′*::mCherry Erdman. 3 w.p.i., lung single-cell suspensions were generated as described above (two mice/sample; *n* = 3; total six mice/drug) and CD64^+^MerTK^+^-infected macrophages were sorted based on the level of expression of CD11c (Fig. S4 f). Sorted CD11c^low^ and CD11c^high^ infected macrophages were then pelleted and resuspended in 100 μl of DMEM supplemented with 10% FBS, 2 mM L-glutamine, and 1 mM sodium pyruvate, and an equal volume of cells for each population was split and seeded into a 96-well plate. Cells were then treated with either 1 μg/ml of INH or RIF or an equal amount of DMSO. 36 h after treatment, cells were lysed and plated on 7H10 agar media for CFU enumeration.

### Data analysis

#### Processing of single-cell datasets

##### Data acquisition and QC

Raw sequencing reads from each run were processed using the software CellRanger (v. 3.0) from 10X Genomics for the mRNA libraries and CITE-Seq-Count (v. 1.4.3; https://hoohm.github.io/CITE-seq-Count/) for the ADT and HTO libraries, to generate raw count matrices for both mRNA and proteins.

Downstream analysis of the datasets was performed in Seurat (v. 3.1.4) as previously described (Stuart et al., 2019). Briefly, we first filtered out cells with <200 unique genes/cell and with very high mitochondrial content (>30% mitochondrial reads). Then multiplexed samples were demultiplexed using the MULTIseqDemux function in Seurat (McGinnis et al., 2019), doublets and empty droplets were identified and removed, and the correct identity was assigned to each sample.

##### Data integration and analysis

scRNA-seq datasets for each sample were preprocessed using the regularized negative binomial regression in Seurat (regressing out both the number of counts and percentage of mitochondrial reads; Hafemeister and Satija, 2019) and analyzed to identify myeloid cell subsets. Myeloid cell subsets from the different samples were then merged to generate an annotated object containing information from all the different datasets. Subsequently, the RNA slot (containing the raw counts) of the merged object was used as an input for Harmony, as previously described (Korsunsky et al., 2019). Briefly, raw counts were log-normalized, and the first 3,000 highly variable genes were identified. The expression of these genes was scaled and centered, PC analysis (PCA) was computed on the scaled expression values, and data integration with Harmony was performed using both “Batch” and “Infection Status” as covariates for the murine datasets and “Batch” for the human datasets. The aligned Harmony embeddings were then used to perform graph-based cluster detection using PCs that were identified as statistically significant by the jackstraw method (Chung and Storey, 2015). The Louvain algorithm was used for community detection (Blondel, 2008). We annotated clusters of cell types by reference-based and canonical marker genes analysis, as described in the text. Trajectory and pseudotime analysis was performed in Monocle (v3.0), using the SeuratWrappers package in R (v. 0.2.0) to convert the integrated Seurat object. For unbiased trajectory and pseudotime analysis of the macrophage populations in both murine and human datasets, all cells previously classified as macrophages were assigned to the same partition, and trajectory/pseudotime analysis was conducted as previously described (Qiu et al., 2017).

Pathway enrichment analysis was performed using G::profiler. For each comparison, we created an ordered by FC list of genes as a query, selecting only those genes where adjusted P value (p-adj) <0.05. The analysis was performed using the g:SCS method for multiple testing correction, the Reactome database as a data source, and the default settings for the other parameters in G::profiler. Only pathways enriched with FDR <0.05 were considered statistically significant.

Manual exploration of the gene lists for each comparison has also been performed to identify relevant themes for genes whose function is described in the literature (e.g., iron genes). For this purpose, we only considered genes whose FC was absolute >1.5 and p-adj <0.05.

### Data analysis for the ATAC-seq datasets

Paired-end sequencing reads of length 2 × 42 bp were trimmed to remove adapter sequences using cutadapt (ver. 2.5; Martin, 2011) and quality-checked using FASTQC. The reads were aligned to the mm10 (GRCm38) mouse reference genome using bwa (ver. 0.7.17; Li and Durbin, 2009). Low-quality alignments (mapping quality < 30), secondary alignments, unmapped reads, and reads with unmapped mates were discarded using samtools. For reads with multiple alignments, only the five best alignments were retained. PCR duplicates were removed using Picard MarkDuplicates. The remaining clean alignments were analyzed for fragment length distribution using the ATACseqQC R/Bioconductor library (Ou et al., 2018). Read alignments to the positive strand were shifted 4 bp downstream, and alignments to the negative strand were shifted 5 bp upstream to center the reads on the transposon binding events. Subsequently, peaks were called using macs2 (ver. 2.1.1.20160309) callpeak command with the parameters “-p 0.01 --shift -75 --extsize 150 --nomodel -B --SPMR --keep-dup all --call-summits.” Fold enrichment and P value tracks normalized to input were produced using macs2 bdgcmp command (options -m FE and -m ppois, respectively). Peaks with a q value <0.01 were shortlisted. Peaks in the blacklisted regions specified by the Encyclopedia of DNA Elements project were removed. For each of the four sample groups, uninfected AM, infected AM, uninfected IM, and infected IM, pooled samples were generated by pooling together reads from all individual replicates of the same group. These pooled samples were also analyzed in the same manner as above. Peaks called in the pooled samples were compared against peaks called in the individual samples of the same condition using the irreproducible discovery rate (IDR) framework. High-quality reproducible peaks were shortlisted based on IDR < 0.05. Subsequently, the IDR peaks from the four groups were merged using “bedtools merge” to generate a final set of peaks (*n* = 127,513). Counts of reads aligned at each peak interval in each sample were determined using the summarizeOverlaps function of the GenomicAlignments R/Bioconductor package (Lawrence et al., 2013). The counts were loaded into edgeR (Robinson et al., 2010) for differential accessibility analysis, where a negative binomial generalized linear model extended with quasi-likelihood methods was fitted to the counts data, dispersions were estimated, and differential peak intensity across sample groups was determined by the quasi-likelihood F-test method (Lun et al., 2016). Visualizations were generated using trimmed mean of M-values normalized and log_2_ transformed counts.

### Data analysis for the bacterial transcriptomes

Data analysis for the Mtb datasets was performed as previously described (Pisu et al., 2020a; Pisu et al., 2020b). Briefly, low-quality reads and Illumina adapters were removed using FlexBar (v. 3.4; Roehr et al., 2017), while remaining rRNA reads were removed using Bowtie2 (-sensitive mode; Langmead and Salzberg, 2012) and a custom GTF file. The filtered fastq files were split using Bowtie2 (–very-sensitive mode) into species-specific files using the two reference genomes, GRCm38.94 for *Mus musculus* and National Center for Biotechnology Information assembly GCA_00668235.1 for Mtb Erdman. Hisat2 (v. 2.1.0; Kim et al., 2015) was then used to align Mtb reads to the Mtb transcriptome, and raw read counts for each sample were obtained using HTSeq (v. 0.11.0; Anders et al., 2015). The raw read count matrices obtained in this study (*hspx′*::GFP^high^ and *hspx′*::GFP^low^) were then combined with the matrices obtained from samples of a previous study (Mtb in AM and Mtb in IM; Pisu et al., 2020a) to compare bacterial responses belonging to ontogeny versus activation of the host immune cell. Exploratory, visualization, and differential gene expression analysis (DGE) was then performed in R using DESeq2 and APEGLM for log FC estimation, as described (Pisu et al., 2020a). Genes with <10 raw counts across all samples were excluded from downstream analysis.

### Statistical analysis

#### scRNA-seq

Differential expression analysis was performed using the nonparametric Wilcoxon rank-sum test as implemented in Seurat (Stuart et al., 2019). Only genes with FDR <0.05 between two comparisons were considered statistically significant. Data integration and batch effect removals were performed with Harmony as previously described (Korsunsky et al., 2019). ADT and HTO data were normalized using a centered log ratio transformation, implemented in the function NormalizeData with normalization.method = ‘‘CLR,’’ in Seurat. RNA counts were log-normalized and scaled before PCA and data integration with Harmony. Data visualizations were generated on the log-normalized counts for the feature plots, scatter plots, and violin plots. Heatmaps and dot plot charts were generated on the scaled expression data, as per default in Seurat.

#### ATAC-geq

Differential accessibility analysis was performed fitting a binomial generalized linear model extended with quasi-likelihood methods to the counts data (Robinson et al., 2010). Differential peak intensity across sample groups was determined by the quasi-likelihood F-test method (Lun et al., 2016).

#### Mtb transcriptome

Statistical testing for the DGE was performed as described (Love et al., 2014). Shrinkage of effect sizes (log FC estimates) has been performed using the APEGLM method (Zhu et al., 2019). Unless specified otherwise, genes having an FDR <0.05 and a FC >1.5 were considered significant. Visualization and clustering were performed on variance stabilized counts (Anders and Huber, 2010) with the option ‘‘blind = TRUE’’ in the DESeq2 package in order to compare samples in an unbiased manner. Heatmaps for specific groups of genes were generated using the normalized counts obtained from the DESeq2 analysis, which have been log-transformed and Z-scaled using the package heatmap2 in R.

#### Flow cytometry analysis of CD11c^high^ and CD11c^low^ subpopulations

MFI of the GFP signal for the CD11c^high^ and CD11c^low^ subpopulation was calculated using the software FlowJo (v. 10.7). Statistical significance was calculated in Prism (GraphPad), performing one-way ANOVA and using the Tukey test correction for multiple comparisons.

#### Drug tolerance

CFU survival data were normalized to 100,000 untreated bacteria. Statistical significance of the difference in mean between the two populations was calculated in Prism (GraphPad) using the unpaired *t* test.

### Online supplemental material

Fig. S1 shows the flow cytometry sorting gates for the infected, bystander, and uninfected mouse samples, confocal imaging of the hspx^high^ and hspx^low^ samples, and QC plots showing the effect of the data integration process on the relative embeddings. Fig. S2 shows flow cytometry analysis for the AM and IM populations in infected and uninfected mouse lung, results of the pathway enrichment analysis for the IM_3 and IM_2 populations, and umap expression plots for different genes. Fig. S3 shows umap expression plots for the RNA and ADT levels of CD11c, CD38, CD11b, and CD14 genes and proteins. Fig. S4 shows the result of pathway enrichment analysis for the AM1_Pro-Infl population, heatmaps for Mtb pathways, and flow cytometry sorting gates for the different Cd11c macrophage populations. Fig. S5 shows various QC metrics for the ATAC-seq datasets and differential peak intensity for a subset of genes highlighted in the text. Table S1 shows the number and percentage of myeloid cells recovered for each infected cell dataset. Table S2 is a DGE results table comparing infected IM_2 versus IM_3 clusters. Table S3 shows marker genes that define the infected IM_2 subpopulation when compared with the rest of the infected cells in the dataset. Table S4 shows marker genes that define the infected IM_3 subpopulation when compared with the rest of the infected cells in the dataset. Table S5 is a DGE results table comparing infected IM_1 versus IM_3 clusters. Table S6 shows marker genes that define the infected IM_1 subpopulation when compared with the rest of the infected cells in the dataset. Table S7 shows marker genes that define the IM_4 subpopulation across all cells, independently of infection status. Table S8 is a DGE results table comparing infected AM_Pro-Infl versus AM_1 clusters. Table S9 shows marker genes that define the infected AM_2 subpopulation when compared with the rest of the infected cells in the dataset. Table S10 is a DGE results table comparing infected AM_2 versus AM_1 clusters. Table S11 is a DGE results table comparing infected AM_2 versus AM_3 clusters. Table S12 is a DGE results table comparing infected AM_2 versus AM_Pro-Infl clusters. Table S13 shows marker genes that define the uninfected AM_2 subpopulation when compared with the rest of the uninfected cells in the dataset. Table S14 is a DGE results table comparing uninfected AM_2 versus AM_3 clusters. Table S15 is a DGE results table comparing uninfected AM_2 versus AM_1 clusters. Table S16 is a DGE results table comparing bystander AM_2 versus AM_1 clusters. Table S17 is a DGE results table comparing bystander AM_2 versus AM_3 clusters. Table S18 shows marker genes that define the bystander AM_2 subpopulation when compared with the rest of the bystander cells in the dataset. Table S19 shows marker genes that define the AM_4 subpopulation across all cells, independently of infection status. Table S20 shows marker genes that define the AM_1 subpopulation across all cells, independently of infection status. Table S21 shows marker genes that define the AM_3 subpopulation across all cells, independently of infection status. Table S22 is a DGE results table comparing Mtb *hspx′*::GFP^low^ versus *hspx′*::GFP^high^ bacteria. Table S23 shows marker genes that define the human_AM_4 subpopulation when compared with the rest of the cells in the dataset. Table S24 shows marker genes that define the human_AM_1 subpopulation when compared with the rest of the cells in the dataset. Table S25 shows marker genes that define the human_AM_2 subpopulation when compared with the rest of the cells in the dataset. Table S26 shows marker genes that define the human_IM_1 subpopulation when compared with the rest of the cells in the dataset. Table S27 shows marker genes that define the human_AM_IM1 subpopulation when compared with the rest of the cells in the dataset. Table S28 shows marker genes that define the human_AI subpopulation when compared with the rest of the cells in the dataset. Table S29 shows marker genes that define the human_AM_MT subpopulation when compared with the rest of the cells in the dataset. Table S30 shows marker genes that define the human_AM_2_exclusive subpopulation when compared with the rest of the cells in the dataset. Table S31 is a DGE results table comparing IM_3 cells in bystander versus infected. Table S32 is a DGE results table comparing bystander IM_2 versus IM_3 clusters. Table S33 is a DGE results table comparing IM_2 cells in bystander versus infected. Table S34 shows ATAC-Seq 7,815 DA peaks comparisons.

## Supplementary Material

Table S1 shows the number and percentage of myeloid cells recovered for each infected cell dataset.

Table S2 is a DGE results table comparing infected IM_2 versus IM_3 clusters.

Table S3 shows marker genes that define the infected IM_2 subpopulation when compared with the rest of the infected cells in the dataset.

Table S4 shows marker genes that define the infected IM_3 subpopulation when compared with the rest of the infected cells in the dataset.

Table S5 is a DGE results table comparing infected IM_1 versus IM_3 clusters.

Table S6 shows marker genes that define the infected IM_1 subpopulation when compared with the rest of the infected cells in the dataset.

Table S7 shows marker genes that define the IM_4 subpopulation across all cells, independently of infection status.

Table S8 is a DGE results table comparing infected AM_Pro-Infl versus AM_1 clusters.

Table S9 shows marker genes that define the infected AM_2 subpopulation when compared with the rest of the infected cells in the dataset.

Table S10 is a DGE results table comparing infected AM_2 versus AM_1 clusters.

Table S11 is a DGE results table comparing infected AM_2 versus AM_3 clusters.

Table S12 is a DGE results table comparing infected AM_2 versus AM_Pro-Infl clusters.

Table S13 shows marker genes that define the uninfected AM_2 subpopulation when compared with the rest of the uninfected cells in the dataset.

Table S14 is a DGE results table comparing uninfected AM_2 versus AM_3 clusters.

Table S15 is a DGE results table comparing uninfected AM_2 versus AM_1 clusters.

Table S16 is a DGE results table comparing bystander AM_2 versus AM_1 clusters.

Table S17 is a DGE results table comparing bystander AM_2 versus AM_3 clusters.

Table S18 shows marker genes that define the bystander AM_2 subpopulation when compared with the rest of the bystander cells in the dataset.

Table S19 shows marker genes that define the AM_4 subpopulation across all cells, independently of infection status.

Table S20 shows marker genes that define the AM_1 subpopulation across all cells, independently of infection status.

Table S21 shows marker genes that define the AM_3 subpopulation across all cells, independently of infection status.

Table S22 is a DGE results table comparing Mtb *hspx′*::GFP^low^ versus *hspx′*::GFP^high^ bacteria.

Table S23 shows marker genes that define the human_AM_4 subpopulation when compared with the rest of the cells in the dataset.

Table S24 shows marker genes that define the human_AM_1 subpopulation when compared with the rest of the cells in the dataset.

Table S25 shows marker genes that define the human_AM_2 subpopulation when compared with the rest of the cells in the dataset.

Table S26 shows marker genes that define the human_IM_1 subpopulation when compared with the rest of the cells in the dataset.

Table S27 shows marker genes that define the human_AM_IM1 subpopulation when compared with the rest of the cells in the dataset.

Table S28 shows marker genes that define the human_AI subpopulation when compared with the rest of the cells in the dataset.

Table S29 shows marker genes that define the human_AM_MT subpopulation when compared with the rest of the cells in the dataset.

Table S30 shows marker genes that define the human_AM_2_exclusive subpopulation when compared with the rest of the cells in the dataset.

Table S31 is a DGE results table comparing IM_3 cells in bystander versus infected.

Table S32 is a DGE results table comparing bystander IM_2 versus IM_3 clusters.

Table S33 is a DGE results table comparing IM_2 cells in bystander versus infected.

Table S34 shows ATAC-seq 7,815 DA peaks comparisons.

## Data Availability

The datasets supporting the conclusions of this study are available in the Gene Expression Omnibus under accession no. GSE167232.
