# Supplementary material for: Single cell analysis of M. tuberculosis phenotype and macrophage lineages in the infected lung
Source: J Exp Med. 2021 Jul 22;218(9):e20210615. doi: 10.1084/jem.20210615 (PMC8302446; doi:10.1084/jem.20210615)
Supplement: Table S1 — shows the number and percentage of myeloid cells recovered for each infected cell dataset. [file JEM_20210615_TableS1.docx]

Table S1. Number and percentage of myeloid cells recovered for each infected cell dataset

| **Sample** | **Total number of cells** | **Number of myeloid cells** | **% of myeloid cells** |
| --- | --- | --- | --- |
| Infected 1 | 4,307 | 2,846 | 66.07% |
| Infected 2 | 5,785 | 4,811 | 83.16% |
| Infected 3 | 3,417 | 2,857 | 83.6% |
| Total | 13,509 | 10,514 | 77.8% |
